# Alterations in voltage-sensing of the mitochondrial permeability transition pore in ANT1-deficient cells

**DOI:** 10.1038/srep26700

**Published:** 2016-05-25

**Authors:** Judit Doczi, Beata Torocsik, Andoni Echaniz-Laguna, Bénédicte Mousson de Camaret, Anatoly Starkov, Natalia Starkova, Aniko Gál, Mária J Molnár, Hibiki Kawamata, Giovanni Manfredi, Vera Adam-Vizi, Christos Chinopoulos

**Affiliations:** 1 Department of Medical Biochemistry, Semmelweis University MTA-SE Laboratory for Neurobiochemistry, Budapest, 1094, Hungary; 2 MTA-SE Lendület Neurobiochemistry Research Group, Budapest, Hungary; 3 Département de Neurologie, Hôpitaux Universitaires, Hôpital de Hautepierre, 67098 Strasbourg cedex, France; 4 Service des Maladies Héréditaires du Métabolisme, Centre de Biologie et de Pathologie Est, CHU Lyon, 69677 Bron cedex, France; 5 Feil Family Brain and Mind Research Institute, Weill Cornell Medical College, New York, NY 10065, USA; 6 Icahn School of Medicine at Mount Sinai, Department of Hematology and Medical Oncology, New York, NY 10029, USA; 7 Institute of Genomic Medicine and Rare Disorders, Semmelweis University, Budapest, 1083, Hungary

## Abstract

The probability of mitochondrial permeability transition (mPT) pore opening is inversely related to the magnitude of the proton electrochemical gradient. The module conferring sensitivity of the pore to this gradient has not been identified. We investigated mPT’s voltage-sensing properties elicited by calcimycin or H_2_O_2_ in human fibroblasts exhibiting partial or complete lack of ANT1 and in C2C12 myotubes with knocked-down ANT1 expression. mPT onset was assessed by measuring *in situ* mitochondrial volume using the ‘thinness ratio’ and the ‘cobalt-calcein’ technique. De-energization hastened calcimycin-induced swelling in control and partially-expressing ANT1 fibroblasts, but not in cells lacking ANT1, despite greater losses of mitochondrial membrane potential. Matrix Ca^2+^ levels measured by X-rhod-1 or mitochondrially-targeted ratiometric biosensor 4mtD3cpv, or ADP-ATP exchange rates did not differ among cell types. ANT1-null fibroblasts were also resistant to H_2_O_2_-induced mitochondrial swelling. Permeabilized C2C12 myotubes with knocked-down ANT1 exhibited higher calcium uptake capacity and voltage-thresholds of mPT opening inferred from cytochrome c release, but intact cells showed no differences in calcimycin-induced onset of mPT, irrespective of energization and ANT1 expression, albeit the number of cells undergoing mPT increased less significantly upon chemically-induced hypoxia than control cells. We conclude that ANT1 confers sensitivity of the pore to the electrochemical gradient.

Mitochondria that are subject to calcium overload exhibit a permeability transition mediated by a pore forming in the inner mitochondrial membrane^1^,^2^. The identity of the structural components of this pore has been until recently unknown; in the past three years though, the c-rings of the ATP synthase^3^,^4^, and the interface within ATP synthase dimers^5^ are being strongly favoured for filling this gap of knowledge^6^,^7^,^2^, but still with a number of questions unanswered, reviewed in^8^,^9^,^10^. Nonetheless, over the past three decades extensive amount of efforts have focused on the regulation and functional characteristics of this phenomenon^11^, and an inverse correlation of the probability of pore opening to the magnitude of the electrochemical gradient has been thoroughly characterized^12^,^13^,^14^,^15^. The molecular entity responsible for the voltage-dependence of the pore has not been identified, though the tuning of this sensor by the oxidation-reduction state of vicinal thiols as well as the contribution of critical arginines has been reported^16^,^17^,^18^,^19^. Relevant to this, glutathione depletion in cultured neurons by monochlorobimane was shown to initiate bioenergetic deficiency that was mediated by inhibition of ANT^20^. ANT exhibits a number of thiols that are amenable to oxidation by several agents^21^,^22^, some of which are unmasked in an energy-dependent manner^23^. Strong circumstantial evidence led to the formulation of the theory that the ANT is a structural part of the pore^24^, backed by hundreds of publications showing impacts of all known ANT ligands on pore opening probability^25^.

However in 2004, the Wallace’s group showed that in the livers of genetically modified mice in which both isoforms of ANT were inactivated, mitochondria still exhibited Ca^2+^-induced pore opening albeit requiring higher amounts of Ca^2+^ loading, and that the effects of ANT ligands were completely abolished^26^. From this report alone, the ANT was deemed as modulatory but not structural component of the mPT. On the other hand, mitochondria obtained from the crustacean *Artemia franciscana* do not exhibit a calcium-regulated pore in view of profound calcium storage^27^, an organism that was recently shown to expresses a unique ANT being refractory to bongkrekic acid^28^, a dual inhibitor for ANT and mPT^29^. Yet, ANTs expressed in related crustacean organisms that lack a Ca^2+^-induced mPT, exhibited sensitivity of ANT-mediated adenine nucleotide exchange to bongkrekic acid^30^, while allogenic expression of Artemia ANT in yeasts also conferred sensitivity to this poison^31^.

Mindful of the above, one may conclude that the ANT is not a structural element of the pore, however, the involvement of this transporter in the regulation of the mitochondrial permeability transition is more than likely. Hereby we investigated the response of *in situ* mitochondria in intact cells lacking partially or completely ANT1 to Ca^2+^-induced mPT opening conferred either by i) the Ca^2+^-ionophore calcimycin or ii) H_2_O_2_, and as a function of the proton electrochemical gradient, also distinguishing between high- *vs* no electron flow in the respiratory chain. We also investigated the voltage thresholds of inducing mPT by submaximally loading permeabilized cells with calcium, and titrating mitochondrial membrane potential with cyanide or an uncoupler. Our results show that ANT1 is the voltage-sensor of the mPT, in line with earlier firm evidences proposing ANT as the voltage-sensor of the pore, as well as being the site of action of oxidative stress^24^,^32^,^33^.

## Results

### Effect of loss of ANT1gene expression on *in situ* mitochondrial swelling induced by calcimycin in human fibroblasts at different bioenergetic states

**Figure 1:**
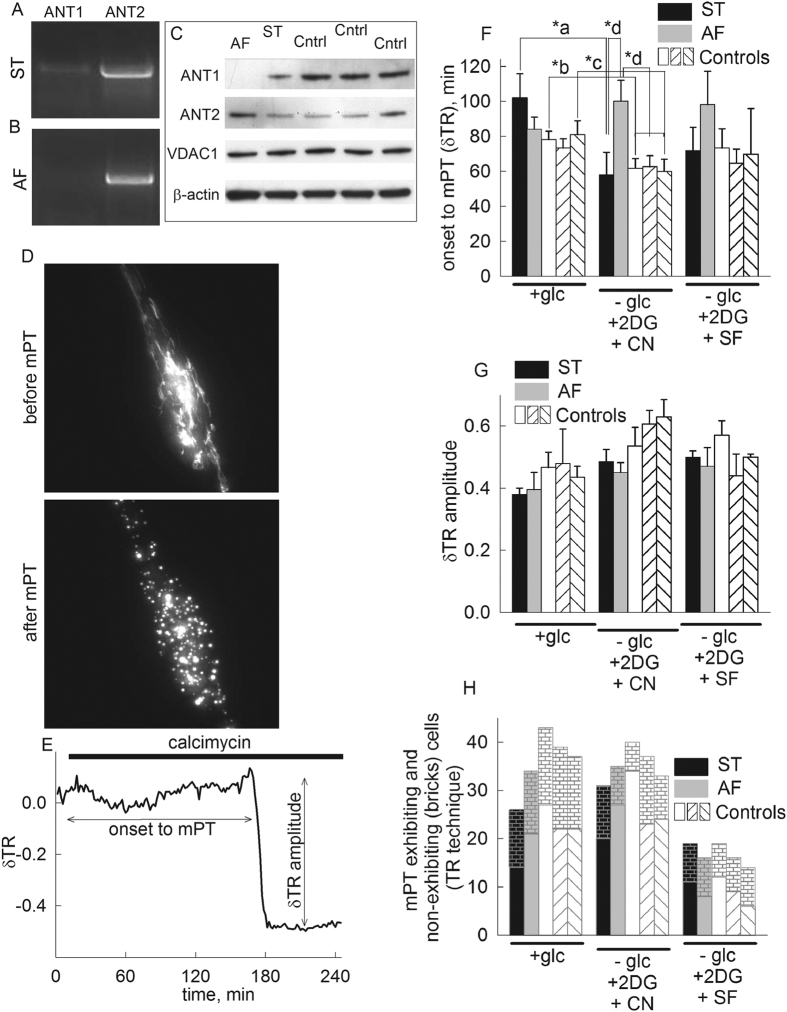
Effect of loss of *ANT1* gene expression on *in situ* mitochondrial swelling (visualized by DsRed2) induced by calcimycin in human fibroblasts during various metabolic conditions [+ glucose, no glucose + 2-deoxyglucose (2 mM) + NaCN (5 mM), no glucose + 2-deoxyglucose + SF 6847 (SF, 1 μM), detailed in the y-axis of the panels]. (**A**) Northern blot image of ST cells for ANT1 and ANT transcripts. (**B**) Northern blot image of AF cells for ANT1 and ANT2 transcripts. (**C**) Scanned images of Western blots of AF, ST and control cells from three healthy donors (35 μg loading per lane) directed against ANT1, ANT2, VDAC1 and β-actin. (**D**) Epifluorescence image of a fibroblast transfected with DsRed2 before and after induction of mPT. (**E**) Representative trace of the effect of calcimycin (2 μM) on the thinness ratio (δTR) indicating mitochondrial volume as a function of time. (**F**) Time elapsed between calcimycin application and mitochondrial swelling (onset to mPT) detected by wide field imaging of mito-DsRed2 expressed in cultured fibroblasts from the heterozygous mother (ST, black bars) the patient (AF, grey bars), and three control subjects (white bars) during various metabolic conditions, detailed under the x-axis. The onset to mPT was determined by the sudden increase of mean mitochondrial diameter in the microscopic view field described as a sudden decrease of the thinness ratio (δTR). Bars indicate means ± S.E.M. of 14–43 cells from at least 8 different cultures as detailed in panel 1D (*p < 0.05 significance by Student’s t-test for ‘a’ ‘b’ and ‘c’ or ANOVA ‘d’; p-values: *a = 0.027, *b = 0.005, *c = 0.04, *d = 0.027. (**G**) Quantification of the δTR amplitude (as marked in panel F) for AF vs ST vs control cells during various metabolic conditions, detailed on the x-axis. (**H**) Comparison of mPT-exhibiting and non-exhibiting cells (bricks) in fibroblasts from ST (black bars), AF (grey bars) and control subjects (white bars) with the TR technique during various metabolic conditions, detailed on the x-axis, using Fisher exact test.

The patient (AF) exhibited a complete loss of *ANT1* expression gene due to a homozygous G to A substitution (c.111 + 1G > A) abolishing the invariable consensus GT splice donor site of intron 1^34^. The patient suffered from cardiomyopathy and myopathy, while her heterozygote mother (ST) was free from these clinical symptoms. In cultured fibroblasts from the patient, mitochondria appeared to be normal under ultrastructural examination and there were no alterations in the activities of respiratory chain complexes; mtDNA rearrangements or increased mtDNA copy number were absent. A thorough description of the clinical case, histopathological and biochemical findings in muscle and cultured fibroblasts appear in ref. 34. Regarding the fibroblasts from the individuals that were used in our study, ST exhibited a faint band of mRNA coding for ANT1, while AF fibroblasts exhibited no band of mRNA coding for this isoform, in northern blotting (panel Fig. 1A,B). mRNA coding for ANT2 appeared to be unaffected (see below under qPCR evaluation). Western blotting analysis (panel Fig. 1C) showed that patient AF had no detectable expression of ANT1, while the heterozygous mother exhibited moderate ANT1 protein expression. On the contrary, ANT2 protein expression was slightly elevated in AF cells compared to either ST cells or the three controls.

Swelling of AF, ST and three control subjects’ mitochondria within fibroblasts were compared during Ca^2+^ overload, induced by addition of calcimycin (4Br-A23187, 2 μM). Although the impact of calcium ionophores on *in situ* mitochondria has not been attributed to the permeability transition in at least one study^35^, there, the effect of the ionophore was not studied for more than 10 minutes. On the other hand, induction of the mPT in *in situ* mitochondria has been succesfully employed in many other cell models, for more extended periods of time^15^,^36^,^37^,^38^,^39^,^40^, as in the present study. The bathing solution of the cells was deprived of Mg^2+^ because calcimycin conducts Ca^2+^ and Mg^2+^ almost equally well. Understandably, mitochondrial swelling in a Ca^2+^-containing but Mg^2+^-free medium may be due to opening of the inner membrane anion channel “IMAC”^41^ and/or K^+^-uniport^42^, in addition to that attributed by the mPT; however, the first two mechanisms operate in all five cell lines, and thus would not serve as confounding factors in the results obtained due to differences in ANT1 expression. Mitochondria were visualized by wide field epifluorescence imaging of either i) mitochondrially targeted DsRed2, or ii) mitochondrially trapped calcein while cytoplasmic calcein fluorescence was quenched by cobalt (see below), a method devised by Petronilli *et al.*^43^. Swelling monitored by evaluating DsRed2-visualized mitochondrial morphology and changes in mean mitochondrial diameters were calculated using the thinness ratio technique, a methodology devised by Gerencser *et al.*^44^. In these assays the onset of swelling was defined by a sudden decrease in the thinness ratio, δTR. A representative epifluorescence image before and after induction of mPT are shown in panel Fig. 1D. A time-lapse series of such experiments are shown in the Supplemental Material, as a function of metabolic conditions (mPT_DsRed2_control_HF_calcimycin_plus_Glc.avi, mPT_DsRed2_control_HF_calcimycin_no_Glc_plus_CN.avi, and mPT_DsRed2_control_HF_calcimycin_no_Glc_plus_UNC.avi). A representative thinness ratio trace is shown in panel Fig. 1E, calculated from the recorded time-series images. In the experiments performed with AF, ST and control subjects’ cells during various metabolic conditions described below, the following parameters were recorded: i) the time elapsed from the addition of calcimycin to the appearance of a large magnitude swelling (marked as ‘onset to mPT’, panel Fig. 1F) ii) the amplitude of swelling (marked as ‘δTR amplitude’, panel Fig. 1G), and iii) the number of cells exhibiting the large magnitude swelling considered as mPT within the experimental time frame (4 hours in the presence of 2 μM calcimycin), depicted in panel Fig. 1H.

As shown in panel Fig. 1F, the calcimycin-induced swelling of *in situ* ST (black bars) mitochondria was hastened by glucose deprivation and NaCN co-application (p-value a^*^ = 0.027, t-test); likewise, swelling of *in situ* mitochondria of two out of the three control subjects’ cells (white bars) was hastened by glucose deprivation and NaCN co-application (p-value: b^*^ = 0.005, t-test and c^*^ = 0.04, t-test). This is consistent with the findings on mouse mitochondria reported previously by our group^15^, and in line with the established phenomenon that a diminished electrochemical gradient primes mitochondria to undergo Ca^2+^-induced mPT. However, the calcimycin-induced swelling of *in situ* AF (grey bars) mitochondria was delayed, compared to other cell types during glucose deprivation and NaCN co-application (p-value: d* = 0.027, one-way ANOVA); furthermore, this parameter was not statistically significantly different from the other cell types during energized conditions. Although the above tendencies were the same for ST and AF cells during glucose deprivation and co-application of the uncoupler SF 6847 compared to the fully energized state, no statistically significant difference was reached.

Within the 4 hours of the experimental time frame the amplitude of swelling (panel Fig. 1G) was not significantly different between ST, AF or control cells under any metabolic condition. This is important, because different cell types exhibit different amplitudes of swelling, for unknown reasons^44^. The absence of a difference in these amplitudes among AF, ST and control cell mitochondria imply that the absence of ANT1 in AF cells did not confer a large, confounding alteration, thus supporting the notion that the mechanism of swelling must be the same for all cells, likely the opening of the permeability transition pore. Accordingly, as shown in panel Fig. 1H, the number of cells exhibiting large-magnitude swelling within the 4 hours of the experimental time frame was also not statistically significant among any cell type.

**Figure 2:**
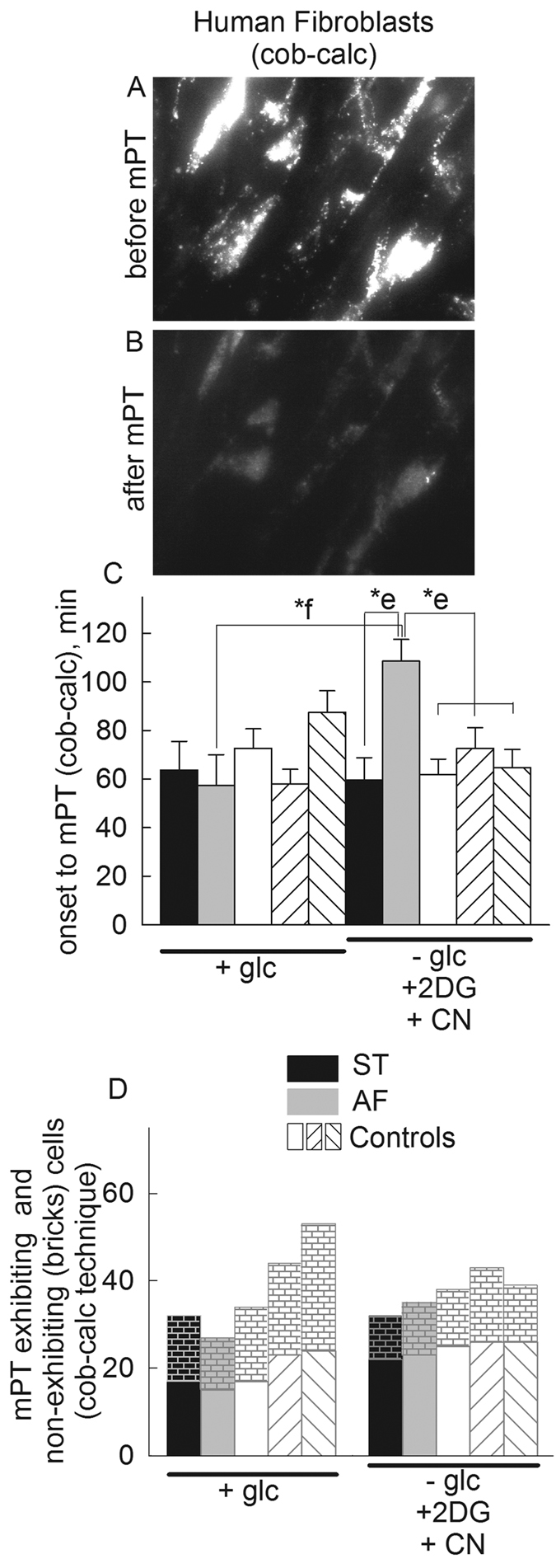
Effect of loss of *ANT1* gene expression on *in situ* mitochondrial swelling (visualized by the cobalt-calcein technique) induced by calcimycin in human fibroblasts during various metabolic conditions [+ glucose, no glucose + 2-deoxyglucose (2 mM) + NaCN (5 mM), detailed in the y-axis of the panels]. (**A**) Epifluorescence image of calcein-loaded fibroblasts before induction of mPT. (**B**) Epifluorescence image of the same fibroblasts as shown in panel A, after induction of mPT. (**C**) Time elapsed between calcimycin application and mitochondrial swelling (onset to mPT) detected by wide field imaging of calcein-loaded cultured fibroblasts from the heterozygous mother (ST, black bars) the patient (AF, grey bars), and three control subjects (white bars) during various metabolic conditions, detailed under the x-axis, in the presence of CoCl_2_. Bars indicate means ± S.E.M. of 29–53 cells from at least 8 different cultures (*p < 0.05 significance by ANOVA; p: *e = 0.002, *f = 0.008; cob-calc: cobalt-calcein technique. (**D**) Comparison of mPT-exhibiting and non-exhibiting cells (bricks) in fibroblasts from ST (black bars), AF (grey bars) and control subjects (white bars) with the cobalt-calcein technique, using Fisher exact test.

Visualization of DsRed2-expressing mitochondria offers superior imaging quality and the fluorescent protein does not exit mitochondria upon swelling. However, transfection efficiency is very low, typically less than 5% in fibroblasts. Therefore, we complemented this approach with the cobalt-calcein technique, a method that outlines the mitochondrial network of all cells. On the other hand, the amount of calcein loading has to be carefully titrated, as it may be toxic to the cells, plus mitochondrially-trapped calcein fluorescence is lost rapidly after opening of the mPT. In our hands, 50 nM of calcein-AM loading at 4 °C was optimal; below this concentration the signal-to-noise ratio was unsatisfactory, while above 100 nM mitochondria from all cell types fragmented a few minutes after loading. Representative epifluorescence images of cells before and after induction of mPT are shown in panel Fig. 2A,B, respectively. A time-lapse series of such an experiment is shown in the Supplemental Material (mPT_COBCA_control_HF_calcimycin_plus_Glc.avi). The thinness ratio technique cannot be applied on calcein-loaded mitochondria, so therefore organellar volume cannot be quantified; using the cobalt-calcein method we only investigated the time elapsing from addition of calcimycin to transformation of mitochondria from a thread-to-ball configuration followed by immediate loss of calcein fluorescence, implying mPT opening. Furthermore, we only investigated the effect of glucose (fully energized state) versus no glucose plus 2-deoxyglucose plus NaCN (fully de-energized state, no electron flow), because the uncoupler SF 6847 was rapidly quenching mitochondrially-trapped calcein fluorescence. The time elapsed until onset of mPT assessed by the cobalt-calcein technique (cob-calc) was less than that assessed by the thinness ratio technique (compare panel Fig. 1F with 2C), in the energized state; this likely reflects the toxicity of loading with calcein. Nevertheless, as shown in panel Fig. 2C, the calcimycin-induced swelling of *in situ* AF (grey bars) mitochondria was delayed, compared to other cell types during glucose deprivation and NaCN co-application (p-value: e^*^ = 0.002, one-way ANOVA). The time elapsed until mPT onset of *in situ* AF (grey bars) mitochondria was also significantly increased in the de-energized compared to the energized state (p-value: f^*^ = 0.008, t-test). The same tendency was observed in the experiments with DsRed2-transfected cells. Similar to the results obtained from DsRed2-expressing mitochondria, as shown in panel Fig. 2D, the number of cells exhibiting loss of mitochondrially-entrapped calcein fluorescence within the experimental time frame was also not statistically significant among any cell type. From the above data we concluded that the absence of ANT1 *increased* the elapsed time to mPT opening upon calcimycin addition only during the de-energized state.

### Effect of complete loss of ANT1 gene expression on matrix Ca^2+^ accumulation upon treatment with calcimycin

To show that application of calcimycin to cells leads to matrix Ca^2+^ accumulation irrespective of the de-energization treatment and to address the possibility that the alterations seen in the elapsed time to mPT upon calcimycin addition between the cell lines (results obtained from the control cell lines were pooled) could be affected by alterations in the accumulation of Ca^2+^ in the mitochondrial matrix, we evaluated matrix Ca^2+^ levels by i) mitochondrially trapped X-rhod-1 (K*d* for Ca^2+^ = 0.7 μM) and ii) genetically encoded, mitochondrially-targeted, ratiometric calcium biosensor that contains enhanced CFP and circularly permuted Venus 173, 4mtD3cpv^45^, (K*d* for Ca^2+^ = 0.6 μM). This FRET-based ratiometric indicator exhibits a nearly exclusive preference for the mitochondrial compartment compared to X-rhod-1 distribution (not shown), though this methodology suffers from low transfection efficiency (typically less than 5%). On the other hand, with X-rhod-1 we could evaluate more cells, but the fluorescence signal could also be partially originating from the cytosolic compartment.

**Figure 3 f3:**
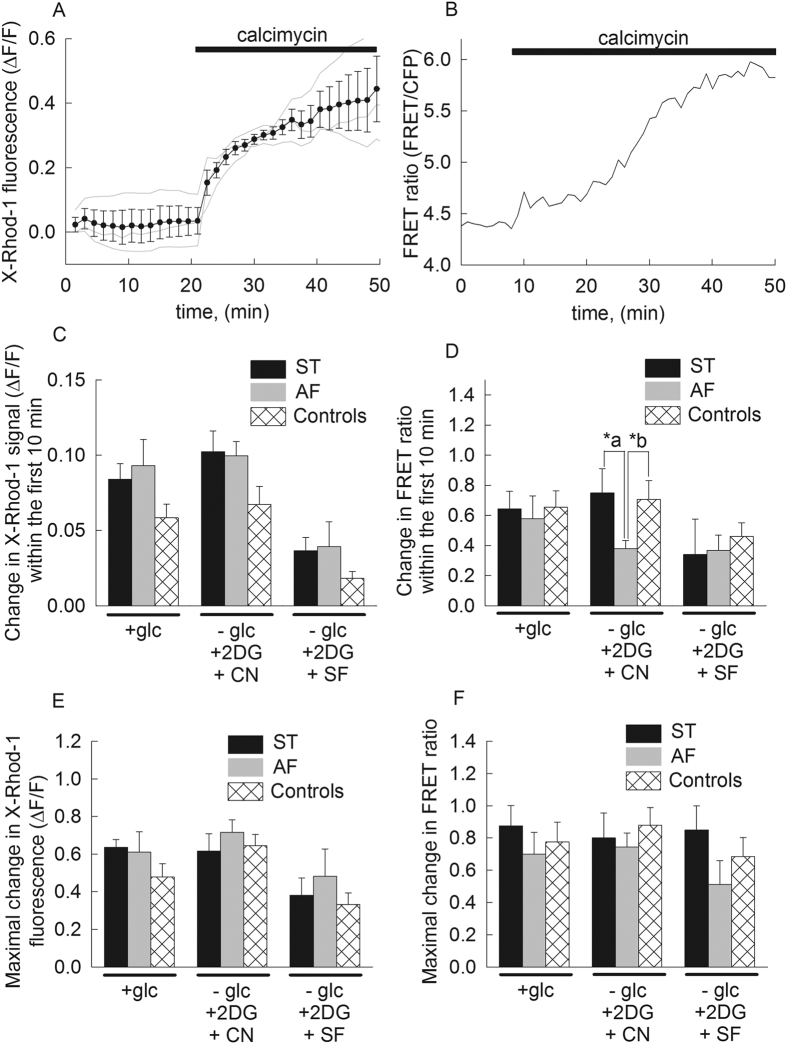
Effect of loss of ANT1 gene expression on matrix Ca^2+^ accumulation upon treatment with calcimycin. (**A**) Representative traces (grey lines) and superimposed mean (black line with S.E.M.) of the effect of calcimycin (2 μM) on mitochondrially-trapped X-Rhod-1 fluorescence, reflecting matrix Ca^2+^ levels. (**B**) Representative trace of the effect of calcimycin (2 μM) on mitochondrially-targeted FRET-based 4mtD3cpv ratio fluorescence, reflecting matrix Ca^2+^ levels. (**C**) Quantification of changes in X-rhod-1 fluorescence within the first 10 min upon addition of calcimycin for various metabolic conditions indicated under the x-axis in cultured fibroblasts from the heterozygous mother (ST, black bars) the patient (AF, grey bars), and three control subjects (pooled cells shown in white hatched bars) during various metabolic conditions, detailed under the x-axis. Bars indicate means ± S.E.M. of 25–40 cells from at least 5 different cultures. (**D**) Quantification of changes in 4mtD3cpv ratio fluorescence within the first 10 min upon addition of calcimycin for various metabolic conditions indicated under the x-axis in cultured fibroblasts from the heterozygous mother (ST, black bars) the patient (AF, grey bars), and three control subjects (pooled cells shown in white hatched bars) during various metabolic conditions, detailed under the x-axis. Bars indicate means ± S.E.M. of 6–10 cells from at least 4 different cultures; p-values: *a = 0.042, *b = 0.044. (**E**) Quantification of maximal changes in X-rhod-1 fluorescence upon addition of calcimycin for various metabolic conditions indicated under the x-axis in cultured fibroblasts from the heterozygous mother (ST, black bars) the patient (AF, grey bars), and three control subjects (pooled cells shown in white hatched bars) during various metabolic conditions, detailed under the x-axis. Bars indicate means ± S.E.M. of 25–40 cells from at least 5 different cultures. (**F**) Quantification of maximal changes in 4mtD3cpv ratio fluorescence upon addition of calcimycin for various metabolic conditions indicated under the x-axis in cultured fibroblasts from the heterozygous mother (ST, black bars) the patient (AF, grey bars), and three control subjects (pooled cells shown in white hatched bars) during various metabolic conditions, detailed under the x-axis. Bars indicate means ± S.E.M. of 6–10 cells from at least 4 different cultures.

Mitochondrial matrix Ca^2+^ accumulation was induced in the various fibroblasts lines in the same manner as for the ‘thinness ratio’, while cells were either loaded with X-rhod-1 (panel Fig. 3A,C,E) or transfected with pcDNA-4mtD3cpv (panel Fig. 3B,D,F). As seen in panel Fig. 3A, representative traces (grey) of X-rhod-1 fluorescence signal representing matrix Ca^2+^ of *in situ* mitochondria are shown from a typical experiment; upon addition of calcimycin (2 μM), there is a gradual elevation in X-rhod-1 fluorescence that reaches a plateau within 30 minutes. The mean trace with S.E.M. bars is shown superimposed in black. In panel Fig. 3B, a representative trace of 4mtD3cpv FRET ratio fluorescence is shown, in response to addition of calcimycin. From such experiments we evaluated matrix Ca^2+^ levels in the various cell lines during the exact metabolic conditions as indicated for panel Fig. 1F, with the following two parameters: i) the rate of change in X-rhod-1 and 4mtD3cpv FRET ratio fluorescence within the first 10 minutes upon addition of calcimycin, and ii) the plateau of X-rhod-1 and 4mtD3cpv FRET ratio fluorescence reached within one hour upon addition of calcimycin. The results of the first parameter are shown in panel Fig. 3C[Fig f3]D and for the second parameter in panel Fig. 3E,F for X-rhod-1 and 4mtD3cpv FRET ratio fluorescence, respectively. All of the results shown in Fig. 3 imply that there is a substantial elevation in matrix Ca^2+^ concentration induced by calcimycin even in the presence of cyanide or uncoupler. However, there was a statistically significant decrease in matrix Ca^2+^ accumulation recorded by 4mtD3cpv FRET ratio fluorescence (panel Fig. 3D) in AF cells compared to ST (p = 0.044) and control fibroblasts (p = 0.042) during de-energization by cyanide. No statistically significant difference was recorded for any other comparison.

### Effect of complete loss of ANT1 gene expression on *in situ* ΔΨm upon treatment with calcimycin

**Figure 4:**
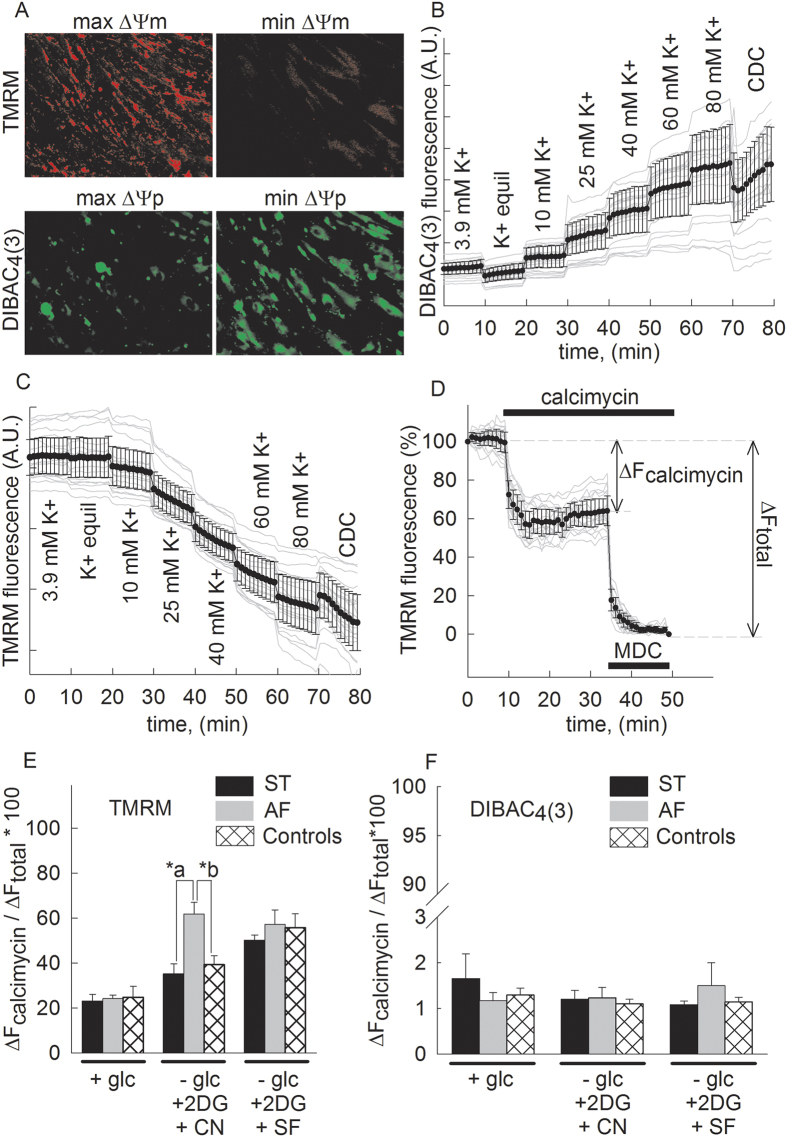
Effect of loss of ANT1 gene expression on *in situ* ΔΨm upon treatment with calcimycin. (**A**) Spectrally un-mixed epifluorescence images of TMRM and DIBAC_4_(3) fluorescence representing ΔΨm and ΔΨp respectively; the methodology of spectral un-mixing is outlined in Supplemental Figs 1 and 2. (**B**) DIBAC_4_(3) fluorescence traces (grey traces) after spectral un-mixing from TMRM fluorescence bleed-through. DIBAC_4_(3) fluorescence intensities (black scatters), indicating mean ± SEM of fluorescence measured over n = 20 fibroblasts (grey lines) in a representative experiment. (**C**) TMRM fluorescence traces after spectral un-mixing from DIBAC_4_(3) fluorescence bleed-through (corresponding DIBAC_4_(3) fluorescence shown in panel B). TMRM fluorescence intensities (black scatters), indicating mean ± SEM of fluorescence measured over n = 20 fibroblasts (grey lines) from the same experiment, shown in panel B. (**D**) Representative traces (grey lines) and superimposed mean (black line with S.E.M.) of the effect of calcimycin (2 μM) on TMRM fluorescence, spectrally un-mixed from DIBAC_4_(3) fluorescence, reflecting ΔΨm. (**E**) Quantification of changes in TMRM fluorescence, spectrally un-mixed from DIBAC_4_(3) fluorescence for the various metabolic conditions indicated under the x-axis, for cultured fibroblasts from the heterozygous mother (ST, black bars) the patient (AF, grey bars), and three control subjects (pooled cells shown in white cross-hatched bars). Bars indicate means ± S.E.M. of 168–227 cells from at least 5 different cultures; p-values: *a < 0.001, *b < 0.001. (**F**) Quantification of changes in DIBAC_4_(3) fluorescence for the various metabolic conditions indicated under the x-axis, for cultured fibroblasts from the heterozygous mother (ST, black bars) the patient (AF, grey bars), and three control subjects (pooled cells shown in white cross-hatched bars). Bars indicate means ± S.E.M. of 168–227 cells from at least 5 different cultures.

As stated above, the threshold of mPT opening depends on the extent of ΔΨm; de-energization by cyanide in the abscence of glucose would lead to reversal of the mitochondrial ATP synthase in an attempt to maintain membrane potential, at the expense of hydrolysing ATP originating from either cytosol (glycolysis) or mitochondrial substrate-level phosphorylation^46^,^47^,^48^,^49^,^50^. Thus, during the de-energization protocol and in the presence of calcimycin, a complete collapse of ΔΨm in our cell lines is not warranted and the presence of a residual potential value is more than likely. To address the status of ΔΨm of the various fibroblast cell lines we measured mitochondrial membrane potential by TMRM and distinguishing it from a plasma membrane distribution signal by co-loading with DiBAC4(3), a negatively charged potentiometric dye. Our method is a variant of that described by Gerencser and colleagues^51^ which used PMPI instead of DiBAC4(3). We used DiBAC4(3) because it exhibits less spectral overlap with TMRM. Representative epifluorescence images of TMRM and DiBAC4(3) fluorescence representing ΔΨm and plasma membrane potential (ΔΨp) respectively, are shown in Supplemental Fig. 1 during addition of the calibrants (see below); image processing flow of the spectral un-mixing performed as described in^52^ is depicted in Supplemental Fig. 2, resulting in the images shown in Fig. 4A. Specifically, the calibration of plasma membrane potential was performed by establishing a K^+^-equilibrium potential (K^+^ equil) at the plasma membrane followed by stepwise increments of extracellular [K^+^] (exact values indicated in panel Fig. 4B,C and Supplemental Fig. 1). The K^+^-equilibrium potential was established by the application of 10 μM diazoxide. Finally, a cell membrane calibration cocktail (CDC) was applied. TMRM signals originating from the mitochondrial compartment was then recorded by spectrally un-mixing the DiBAC4(3) fluorescence signal as described in^51^ using decomposition algorithms developed in^52^.

Representative traces (grey lines) of TMRM fluorescence (spectrally un-mixed from DiBAC4(3) fluorescence, see Supplemental Figs 1 and 2 and details under Materials and Methods) reflecting mitochondrial membrane potential from control fibroblasts under energized conditions are shown in panel Fig. 4D; the mean trace with S.E.M. bars is shown superimposed in black. As shown in panel Fig. 4D, addition of calcimycin decreased TMRM fluorescence by approximately 40%; residual ΔΨm was lost upon further addition of a mitochondrial membrane depolarization cocktail (MDC), the composition of which is detailed under Materials and Methods. From such experiments we evaluated ΔΨm levels in the various cell lines (results obtained from the control cell lines were pooled) during the exact metabolic conditions as indicated for panel Fig. 1F, by taking into account the ratio of calcimycin-induced (ΔF_calcimycin_) to MDC-induced (ΔF_total_) change in TMRM fluorescence. The results are summarized in panel Fig. 4E; as shown in panel Fig. 4E, the mitochondrial de-energization induced by calcimycin -in addition to that by cyanide- is highly significantly (p = 0.001) greater in AF cells compared to either ST or control cells. Yet, the time lag until onset to mPT (shown in panel Fig. 1F) is greater under the same de-energization conditions for the AF cells. These two results are strongly supportive of the notion that lack of ANT1 renders mitochondria insensitive to modulation of mPT opening by the mitochondrial membrane potential. On the other hand, ΔΨp inferred by DiBAC4(3) fluorescence remained relatively unchanged (<2% changes), panel Fig. 4F. The fact that plasma membrane potential does not show large fluctuations, affords greater assurance that the TMRM signals are almost entirely due to fluctuations of the mitochondrial membrane potential, since TMRM is known to distribute across the plasma membrane as well^53^.

### Effect of loss of ANT1 gene expression on *in situ* mitochondrial swelling induced by H_2_O_2_ in human fibroblasts

**Figure 5:**
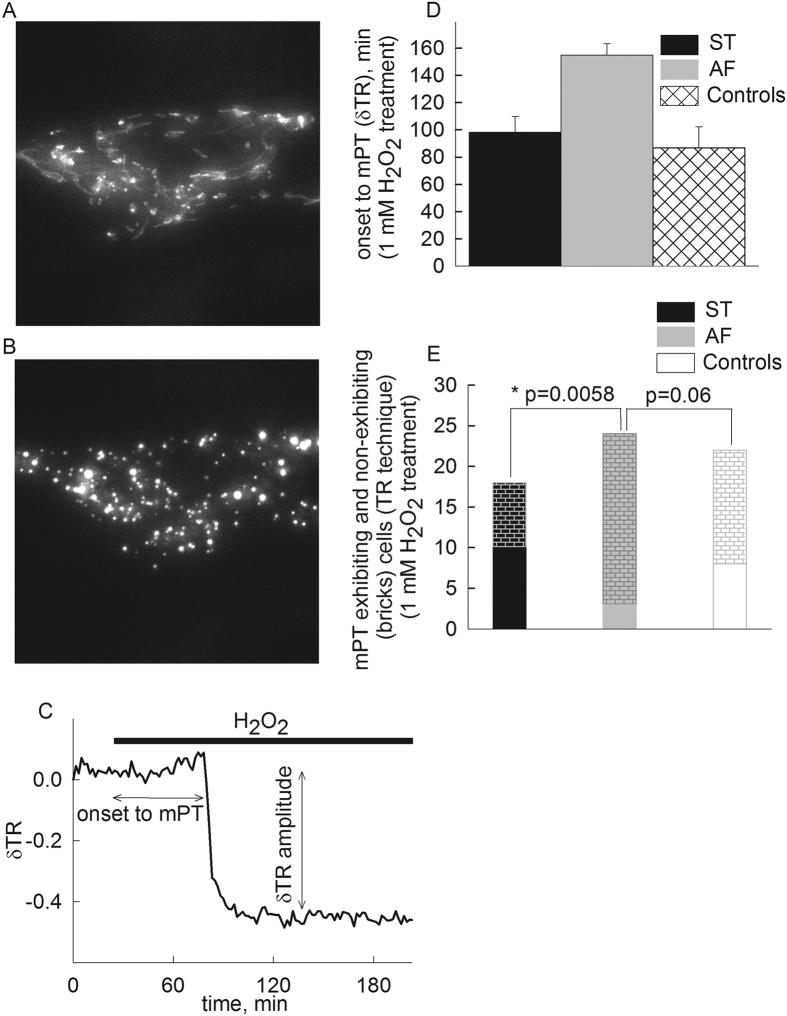
Effect of loss of *ANT1* gene expression on *in situ* mitochondrial swelling (visualized by DsRed2) induced by H_2_O_2_ in human fibroblasts. (**A**) Epifluorescence image of a fibroblast transfected with DsRed2 before induction of mPT. (**B**) Epifluorescence image of the same fibroblast as shown in panel 5A, after induction of mPT by H_2_O_2_. (**C**) Representative trace of the effect of H_2_O_2_ (1 mM) on the thinness ratio (δTR) indicating mitochondrial volume as a function of time. (**D**) Time elapsed between H_2_O_2_ application and mitochondrial swelling (onset to mPT) detected by wide field imaging of mito-DsRed2 expressed in cultured fibroblasts from the heterozygous mother (ST, black bars) the patient (AF, grey bars), and three control subjects (white cross-hatched bars) in the presence of glucose. The onset to mPT was determined by the sudden increase of mean mitochondrial diameter in the microscopic view field described as a sudden decrease of the thinness ratio (δTR). Bars indicate means ± S.E.M. of 18–24 cells from at least five different cultures. (**E**) Comparison of mPT-exhibiting and non-exhibiting cells (bricks) in fibroblasts from ST (black bars), AF (grey bars) and control subjects (white bars) with the TR technique, using Fisher exact test.

To address the role of ANT1 on mPT induced by a different than Ca^2+^-overload stimulus, we subjected the fibroblasts to an oxidant treatment by 1 mM H_2_O_2_. Reactive oxygen species are known to induce mPT^54^. Just like with calcimycin, treatment of DsRed2-transfected cells with H_2_O_2_ (without omitting Mg^2+^ from the medium) led to an abrupt change in mitochondrial morphology which by the thinness ratio technique is quantified as swelling, thus implying mPT opening. Representative epifluorescence images of a cell before and after mPT induction are shown in panel Fig. 5A,B, respectively. A time-lapse series of such an experiment is shown in the Supplemental Material (mPT_DsRed2_control_HF_H2O2.avi). A representative thinness ratio trace is shown in panel Fig. 5C, calculated from the recorded time-series images. In the experiments using H_2_O_2_ as an mPT inducer, we did not variate the metabolic conditions by omitting glucose or including cyanide or uncoupler, because H_2_O_2_ is expected to exert a considerable metabolic challenge itself^55^. In AF, ST and pooled control subjects’ cells, the following parameters were recorded: i) the time elapsed from the addition of H_2_O_2_ to the appearance of a large magnitude swelling (marked as ‘onset to mPT’, panel Fig. 5D), and ii) the number of cells exhibiting the large magnitude swelling considered as mPT within the experimental time frame (4 hours in the presence of 1 mM H_2_O_2_), depicted in panel Fig. 5E.

As shown in panel Fig. 5D, the H_2_O_2_-induced onset to mPT of *in situ* ST (black bar) mitochondria was similar to that from the control cells (cross-hatched white bar). However, the H_2_O_2_-induced onset to mPT of *in situ* AF (grey bar) mitochondria was delayed, compared to other cell types. Although this difference is large (~60% further delay in onset to mPT), it was not statistically significantly different from the other cell types but that is only because a very low number of cells (only three) underwent mPT, reducing the power of statistical analysis. Exactly because of this reason, as shown in panel Fig. 5E, the number of AF cells that underwent mPT was much smaller (p = 0.0058) that the other cell types, also leading to not significant differences between AF and control cells evaluated with the Fisher exact test (p = 0.06), but yielded significance using the less stringent Chi-square test (p = 0.029).

From the above data we concluded that the absence of ANT1 may increase the elapsed time to mPT upon H_2_O_2_ addition, but most importantly prevent cells from undergoing mPT. These results are in accordance to those by Kokoszka and colleagues^26^, where they showed that although tert-butyl hydroperoxide, a non-specific oxidant, and diamide, a specific –SH group oxidant, facilitated isolated mouse liver mitochondria to undergo mPT independent of the presence of ANT, ANT1−/− /ANT2−/− deficient mice exhibited more than three times higher maximum Ca^2+^ uptake capacity compared to wild-type mice^26^.

### Effect of knocking-down Ant1 expression in C2C12 myotubes on *in situ* mitochondrial swelling induced by calcimycin at different bioenergetic states

**Figure 6:**
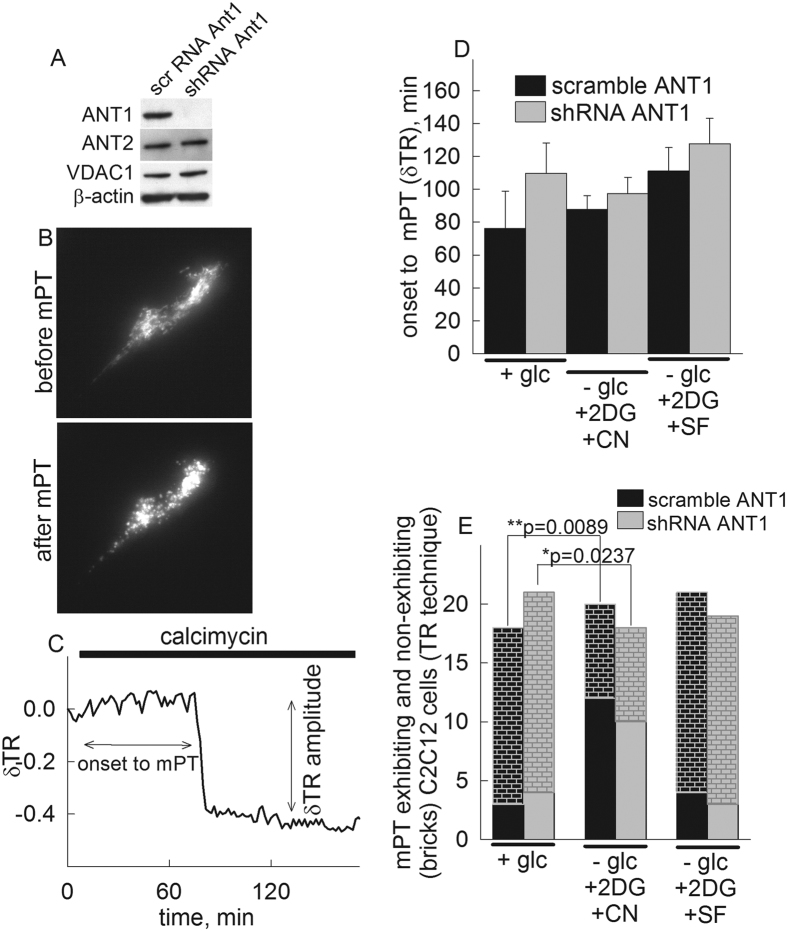
Effect of knocking-down Ant1 in C2C12 myotubes by shRNA using lentiviruses, versus scramble RNA-transfected cells on calcimycin-induced mitochondrial swelling or maximum calcium uptake capacity. (**A**) Scanned images of Western blots of C2C12 myotubes (10 μg loading per lane) transfected with shRNA directed against Ant1 or scramble (scr) RNA, for ANT1, ANT2, VDAC1 and β-actin. (**B**) Epifluorescence image of a C2C12 myotube transfected with DsRed2 before and after induction of mPT. (**C**) Representative trace of the effect of calcimycin (2 μM) on the thinness ratio (δTR) indicating mitochondrial volume as a function of time. (**D**) Time elapsed between calcimycin application and mitochondrial swelling (onset to mPT) detected by wide field imaging of mito-DsRed2 expressed in cultured C2C12 cells treated with shRNA for Ant1 or scramble RNA during various metabolic conditions, detailed under the x-axis. The onset to mPT was determined by the sudden increase of mean mitochondrial diameter in the microscopic view field described as a sudden decrease of the thinness ratio (δTR). Bars indicate means ± S.E.M. of 18–21 cells from at least five different cultures. (**E**) Comparison of mPT-exhibiting and non-exhibiting cells (bricks) detected with the TR technique during various metabolic conditions, using Fisher exact test.

In order to evaluate the role of ANT1 on voltage-sensing properties of the mPT in a different cell context, we knocked-down its gene by stably expressing lentiviral shRNA targeted against mouse Ant1 as performed in^56^, and swelling of *in situ* mitochondria of these versus scramble RNA (scr) transfected cells were compared during Ca^2+^ overload induced by addition of calcimycin, as a function of various metabolic conditions. As in Kawamata *et al.*^56^, ANT1 expression in shRNA-treated cells was undetectable by Western blotting (see panel Fig. 6A), and qPCR for mRNA was reduced by over 90% (not shown). Expression of ANT2 was unaffected, (panel Fig. 6A). *In situ* mitochondria were visualized by wide field epifluorescence imaging of mitochondrially targeted DsRed2. Swelling was monitored by evaluating DsRed2-visualized mitochondrial morphology and changes in mean mitochondrial diameters were calculated using the thinness ratio technique. Representative epifluorescence images before and after induction of mPT are shown in panel Fig. 6B. Time-lapse series of such experiments from an shRNA-transfected and a scramble RNA-transfected C2C12 cell are shown in the Supplemental Material, (mPT_DsRed2_C2C12_shRNA_ANT1.avi, and mPT_DsRed2_C2C12_scramble_ANT1.avi). A representative thinness ratio trace is shown in panel Fig. 6C, calculated from the recorded time-series images. In the experiments performed with shRNA ANT1-transfected and a scramble RNA-transfected C2C12 cells during various metabolic conditions as described in the legend and panels, the following parameters were recorded: i) the time elapsed from the addition of calcimycin to the appearance of a large magnitude swelling (marked as ‘onset to mPT’, panel Fig. 6D) and ii) the number of cells exhibiting the large magnitude swelling considered as mPT within the experimental time frame (4 hours in the presence of 2 μM calcimycin), depicted in panel Fig. 6E. As shown in panel Fig. 6D, the calcimycin-induced onset to mPT in C2C12 cells with knocked-down ANT1 did not exhibit a statistically significant difference from scramble RNA transfected cells, under any metabolic conditions. However, as shown in panel Fig. 6E, the number of cells exhibiting large-magnitude swelling within the 4 hours of the experimental time frame was statistically significantly different among these cell types when comparing glucose deprivation and NaCN co-application versus no glucose deprivation. Nonetheless, the number of C2C12 cells with knocked-down Ant1 undergoing mPT increased less significantly (p = 0.0089 vs p = 0.0237) upon chemically-induced hypoxia than cells transfected with scramble RNA. This observation attests to the notion that ANT1 at least partially regulates mPT opening as a function of energization level of *in situ* mitochondria. However, in this experimental setting and just like in human fibroblasts, de-energization by uncoupler did not confer the same alterations in mPT exhibiting versus non-exhibiting cells.

### Effect of knocking-down Ant1 expression in permeabilized C2C12 myotubes on the voltage threshold of calcium-induced mPT

**Figure 7:**
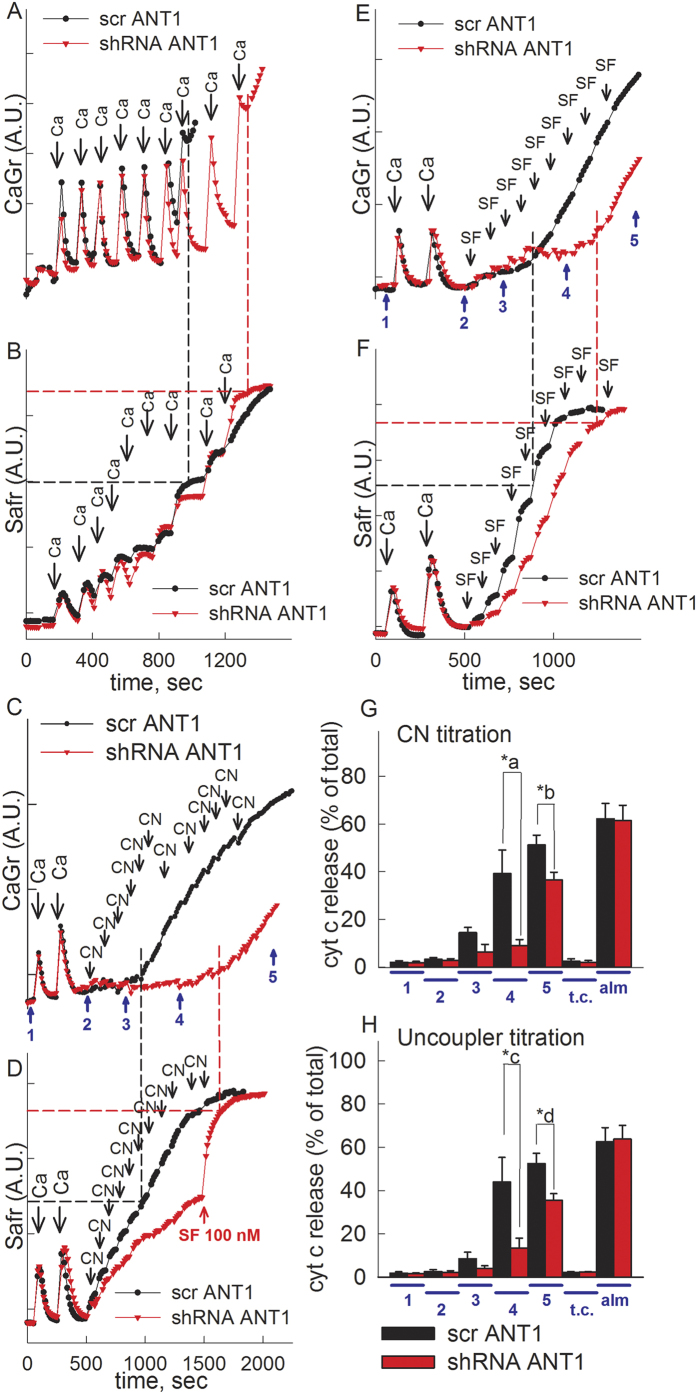
Determination of voltage thresholds of calcium-induced mPT in permeabilized C2C12 cells treated with shRNA for Ant1 (red inverted triangles) or scramble RNA (black closed circles), under various metabolic conditions. (**A**) Maximum calcium uptake capacity in permeabilized C2C12 cells treated with shRNA for Ant1 or scramble RNA. Each addition of CaCl_2_ (Ca) is 100 μM. (**B**) Safranine O fluorescence traces of C2C12 cells treated exactly as in panel A. Mitochondrial substrates were 5 mM glutamate and 5 mM malate. (**C**) Cells were challenged by two additions of CaCl_2_ (Ca, 100 μM each) followed by consecutive additions of 50 μM NaCN, and extramitochondrial calcium was measured by CaGr. Blue numbers and arrows signify time-points of probing for cytochrome c release (see panel **G**). (**D**) Safranine O fluorescence traces of C2C12 cells treated exactly as in panel C, with the exception that shRNA-treated cells received a bolus of 100 nM SF6847 as shown by the red arrow, to indicate that prior to this addition a significant portion of ΔΨm remained. (**E**) Cells were challenged by two additions of CaCl_2_ (Ca, 100 μM each) followed by consecutive additions of 10 nM SF6847, and extramitochondrial calcium was measured by CaGr. Blue numbers and arrows signify time-points of probing for cytochrome c release (see panel H). (**F**) Safranine O fluorescence traces of C2C12 cells treated exactly as in panel E. All traces shown in panels (A-F) are representative of 5 independent experiments. (**G**) Cytochrome c release (% of the total) estimated by enzyme-linked immunosorbent assay from permeabilized C2C12 cells probed at time points (1–5) indicated in panel C; *a = 0.034, *b = 0.049. (**H**) Cytochrome c release (% of the total) estimated by enzyme-linked immunosorbent assay from permeabilized C2C12 cells probed at time points (1–5) indicated in panel E; *c = 0.047, *d = 0.041. ‘Time-controls (t.c.)’ signify probing of C2C12 cells at time-point 5 for cytochrome c release that have not been treated with either CaCl_2_ or NaCN nor SF6847. ‘Alm’ signifies probing of C2C12 cells for cytochrome c release that have been treated with 80 ngr of alamethicin. Bars indicate means ± S.E.M. of ~270,000 cells from 3 different cultures.

We further evaluated the role of ANT1 on the voltage threshold of calcium-induced mPT under various metabolic conditions, in permeabilized C2C12 cells and measuring mitochondrial calcium uptake, ΔΨm, and cytochrome c release using the following protocols: i) maximum calcium uptake of *in situ* mitochondria supported by glutamate and malate (panels 7A and 7B, recording calcium uptake and ΔΨm, respectively), ii) challenge by submaximal CaCl_2_ dose followed by titration with step-wise inhibition of respiration by NaCN (50 μM boluses) shown in panels 7C and 7D, recording calcium uptake and ΔΨm, respectively, iii) challenge by submaximal CaCl_2_ dose followed by titration with step-wise dissipation of ΔΨm by SF6847 (10 nM boluses) shown in panels 7E and 7F, recording calcium uptake and ΔΨm, respectively, and iv) measuring cytochrome c released from the *in situ* mitochondria of the permeabilized cells during the following time points, indicated in panels 7C and 7E: 1 = before calcium additions, 2 = right after calcium additions, 3 = before loss of sequestered calcium and membrane potential, 4 = after loss of sequestered calcium in scramble RNA treated cells but not shRNA treated cells, 5 = after loss of sequestered calcium and membrane potential in both cells types, plus a time control for point 5 (cells were probed for cytochrome c release after 20 min of incubation but were not challenged by either CaCl_2_ or NaCN or uncoupler) and an alamethicin (a pore-forming peptide) control. The amount of cytochrome c appearing in the supernatant upon treatment with alamethicin provides a measure of the maximum amount releasable^57^. Calcium uptake and ΔΨm measurements were performed in parallel but different samples (but from the same cultures) due to spectral overlap of calcium green and safranine O. The assessment of maximum calcium uptake capacity is a widely utilized protocol^58^,^59^, that can be performed in isolated^60^, or *in situ* mitochondria of permeabilized cells. In our hands, permeabilized human fibroblasts exhibited a very small maximum mitochondrial calcium uptake capacity, less than 10 nmol/mg protein, and therefore, experiments with these cells were not conducted. On the contrary, permeabilized C2C12 myotubes exhibited very large maximum calcium uptake capacities, providing the opportunity for a more thorough investigation of mPT opening by calcium loading as a function of various metabolic conditions. We have tried to isolate mitochondria from fibroblasts and C2C12 cells, but there were insurmountable obstacles with both cell types; with fibroblasts, an extremely small amount of isolated mitochondria was obtained, not suitable for functional experiments; with C2C12 cells only very harsh homogenization methods could yield sufficient amount of mitochondria, but they were damaged to the extent that they were too leaky for functional studies. For these reasons, we only evaluated C2C12 cells. shRNA-transfected and scramble RNA-transfected C2C12 cells were permeabilized as described under ‘Materials and Methods’, boluses of 100 μM CaCl_2_ (panel Fig. 7A,B) were added where indicated, and extramitochondrial Ca^2+^ levels were followed by Calcium Green 5N hexapotassium salt fluorescence (Fig. 7A), or ΔΨm, using safranine O (Fig. 7B). As shown in panel Fig. 7A, C2C12 cells with knocked-down Ant1 exhibited larger mitochondrial calcium uptake capacity than scramble-transfected cells, in the presence of glutamate (5 mM) and malate (5 mM). It is to be noted, that this is not a condition in which mitochondria are continuously fully energized; it is well understood that upon progressive mitochondrial calcium sequestration, mitochondria depolarize accordingly^61^. Indeed, as shown in figure panel 7B, ΔΨm of both cell types gradually dissipates upon increasing calcium load. However, as it is marked by black dashed line for scr RNA-treated cells and red dashed line for shRNA-treated cells, those cells with knocked-down ANT1 expression exhibited loss of sequestered calcium in a much more depolarized ΔΨm range, nearing that of complete depolarization. Next, to obtain a more accurate indication of how mPT opening relates to ΔΨm, we challenged *in situ* mitochondria of permeabilized C2C12 cells with an amount of CaCl_2_ that is insufficient to trigger pore opening, and then dissipated ΔΨm gradually by inhibiting complex IV with cyanide (50 μM boluses, Fig. 7C,D) or using the uncoupler SF 6847 (10 nM boluses Fig. 7E,F), while recording extramitochondrial Ca^2+^ levels (Fig. 7C,E), or ΔΨm (Fig. 7D,F). In figure panel 7D the addition of 100 nM SF 6847 is also shown (red arrow), signifying that just prior to its addition there was a considerable extent of ΔΨm that could be dissipated. As shown in Fig. 7C–F and marked by black dashed line for scr RNA-treated cells and red dashed line for shRNA-treated cells, those cells with knocked-down ANT1 expression exhibited loss of sequestered calcium in a much more depolarized ΔΨm range, again nearing that of complete depolarization. Furthermore, as shown in figure panels 7G and 7H, a higher percentage of cytochrome c was released from scr RNA treated cells at time points 4 and 5 compared to that from shRNA treated cells, implying that the loss of ΔΨm and sequestered calcium during the same time frame is due to large amplitude swelling resulting in rupture of the outer mitochondrial membrane, implying mPT opening. From these experiments we concluded that ANT1 regulates the voltage threshold of calcium-induced mPT opening.

### Effect of complete loss of ANT1 gene expression on ADP-ATP exchange rates as a function of ΔΨm

**Figure 8:**
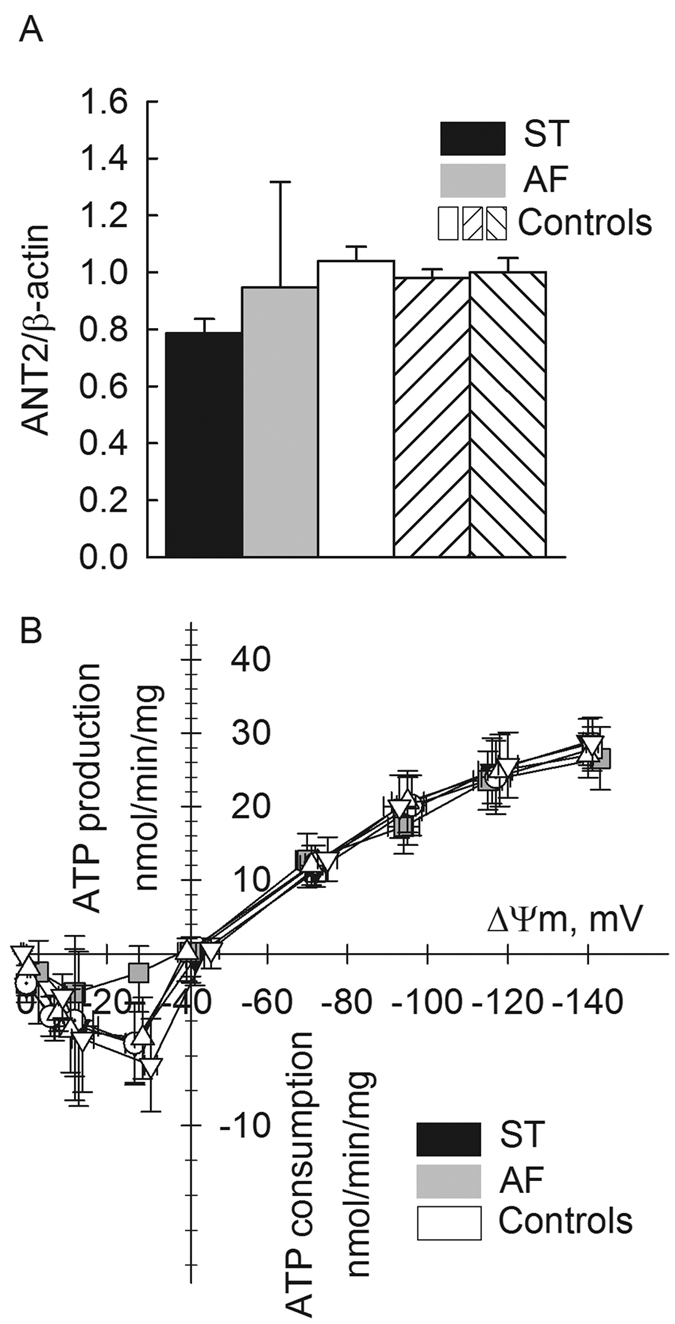
*ANT2* expression and ADP/ATP exchange rates as a function of mitochondrial membrane potential. (**A**) Reverse transcription PCR and amplification of cDNAs reflecting expression of *ANT2* gene in cultured fibroblasts of the patient (AF), the heterozygous mother (ST) and three control subjects, as a ratio to β-actin expression. (**B**) Plot of ATP–ADP exchange rate mediated by ANT versus ΔΨm in *in situ* mitochondria of AF (grey squares), ST (black squares) and control subjects (white symbols) permeabilized cells depolarized to various voltages by increasing amounts of SF 6847; constructed from the data of 3 independent experiments.

Human fibroblasts express three (1, 2 and 3) out of the four known isoforms of ANT. *ANT2* was not evaluated particularly for AF and ST cells in ref. 34, therefore we quantified this transcript by qPCR. As shown in panel Fig. 8A, AF cells did not exhibit a statistically significant increase of the *ANT2* gene expression compared to ST and control subject cells. This and the unchanged levels of *ANT3* expression reported in ref. 34 yielded ADP-ATP exchange rates as a function of ΔΨm that did not differ between AF, ST and control subjects’ cells, shown in panel Fig. 8B. As reported in ref. 34, AF cells did not express *ANT1* (verified in results shown in panel Fig. 1B,C). From these results we concluded that the differences in onset of the calcimycin-induced mPT between AF, ST and control subjects’ cells were unlikely to be attributed to alterations in the adenine nucleotide and/or ΔΨm levels. The determination of the amount of functional ANTs by means of titration with carboxyatractyloside while measuring respiration could not be reliably performed due to the low amount of mitochondria that could be accessed in the permeabilized cultures; this is because the amount of carboxyatractyloside required, was estimated to be in the low nanomolar range, *i.e.* near the K_i_ of the inhibitor for the ANT (not shown). Neither AF nor ST cells exhibited appreciable ANT reversal rates, probably due to the prominent expression of IF-1 in human fibroblasts^62^, also supported by our findings with the cells from three different control subjects, shown in panel figure bB.

## Discussion

mPT is implicated in many human diseases^2^,^63^. Extensive characterization of the phenomenon has assisted in the recognition of certain proteins as regulatory components, such as cyclophilin D and ANT^1^. VDAC was also initially considered as a structural component, however, tissues engineered to lack all known isoforms still exhibited mPT pore opening and regulation, reviewed in^64^. Furthermore, closure of VDAC hastened mPT pore opening by means of impeding the efflux of superoxide anions from the intermembrane space^65^. Likewise, the outer membrane translocator protein of 18 kDa, TSPO, was originally reported to participate in mPT regulation^66^,^67^,^68^, a claim to be recently disproved^69^. Prior to the report that ANT-deficient mice still exhibit the mPT^26^, the ANT was already dismissed as a structural element of the pore in dimer formation induced by pore-inducing oxidants^70^; however, formation of ANT dimers need not materialize for pore assembly. Oxidants and other chemical modifiers are known to modulate the mPT; there are at least three sites for oxidation tuning a sensor that translates the changes of both transmembrane voltage and surface potential into changes of the mPT pore open probability^63^. The critical arginines thought to tune the voltage sensor of the pore were considered not to reside in the ANT^18^,^19^. However, the experiments shown above support the notion that in human fibroblast mitochondria and in C2C12 myotubes the sensor -or at least a part of it- is likely to be isoform 1 of the ANT. Therefore, the sensing property of ANT1 is not mediated through its arginines. The possibility that other oxidation-prone sites tuning the pore sensor reside on ANT1 remains high; indeed, in the presence of oxidants, ANT1^−/−^ /ANT2^−/−^ deficient mice exhibited more than three times higher maximum Ca^2+^ uptake capacity compared to wild-type mice^26^.

Most of the experiments by Bernardi’s group that proved the inverse correlation of the proton electrochemical gradient to mPT pore opening probability were performed with rat liver mitochondria where ANT2 is the dominant isoform mediating ADP/ATP exchanges, however, in rats ANT1 also exhibits significant expression^71^.

ANT1 (but not ANT2) is considered proapoptotic^72^. The proapoptotic property of ANT1 is extremely dominant: overexpression of ANT1 in tumors *in vivo* (where otherwise ANT2 is mostly expressed) induces apoptosis and tumor regression^73^. However, silencing of ANT1 expression in a human glioblastoma cell line induced a paraptotic-like process^74^. On the other hand, overexpression of ANT1 is associated with autosomal dominant facioscapulohumeral muscular dystrophy^75^. In addition, it was shown that mice deficient in ANT1 exhibit increased resistance to excitotoxic insults of the brain^76^. Brain mitochondria from these mice exhibited ~20% higher maximum Ca^2+^ uptake capacity; unfortunately they were not investigated for mPT under de-energized conditions^76^.

In^26^, WT and ANT1^−/−^/ANT2^−/−^ -deficient mouse liver mitochondria responded equally to uncoupler-induced swelling; we also found no statistically significant difference between ST, control and AF cells, even though there was a trend for AF mitochondria to exhibit smaller ‘onset to mPT’ values. As a word of caution though: the ANT1^−/−^/ANT2^−/−^ -deficient mice mitochondria exhibited stark differences in bioenergetic parameters and expression levels of other mitochondrial proteins^26^, likely the result of compensatory mechanisms, while in this study fibroblasts and C2C12 myotubes were not confounded by other bioenergetic alterations, in line with the results obtained in^34^. However, it is interesting that in AF cells there seems to be a rebound upregulation in ANT2 expression. Since this was observed in Western blots but not Northern blots, it is likely to be due to regulation in the posttranslational modification of the ANT2 protein, and/or its degradation pathway. Relevant to this, different voltage-sensing properties of different ANT isoforms is a possibility. We do acknowledge though that to truly isolate ANT1’s functions from possible compensatory effects of other ANT isoforms, re-introduction of expression of ANT isoform 1 in AF cells would prove that only ANT1 is sensitive to alterations of the electrochemical gradient. However, to achieve this, ANT1 level expression should be matched to those observed in the control cell lines, a task posing serious technological challenges.

There is an important difference between de-energization of cells by glucose deprivation and either chemical anoxia, or an uncoupler; in the former, there is no electron flow in the electron transport chain; in the latter, there is high electron flow, despite glucose deprivation due to the presence of endogenous substrates that can fuel the citric acid cycle and yield reducing equivalents. mPT is indeed tuned by a voltage sensor, but in addition to that, electron flow through complex I strongly dictates pore-opening probability^15^,^77^,^78^ and is maybe part of the voltage-sensing mechanism. Furthermore, there is a potential additional confounding factor regarding the action of uncouplers: mitochondrial Ca^2+^ release by uncouplers is usually ascribed to matrix acidification^79^,^80^, which induces dissociation of the Ca^2+^phosphate complex. However, matrix acidification by nigericin in the presence of a complete collapse of ΔΨm by combined inhibition of the respiratory chain and the F_0_-F_1_ ATP synthase, lead to the release of only one-fifth of sequestered calcium^61^,^81^. Thus, uncouplers exert some other, yet to be identified, effect regarding matrix Ca^2+^ release, which may share a common denominator with induction of the permeability transition.

In the present report we show that in *in situ* mitochondria of ANT1-deficient human fibroblast and C2C12 cells with knocked-down ANT1 by a lentiviral approach, the hastening of Ca^2+^-induced mPT pore opening during glucose deprivation and chemical anoxia is reduced or abolished. A similar picture emerged from or H_2_O_2_- treated cells shown to induce mPT opening, with well-documented adverse bioenergetic effects. Finally, voltage-thresholds of calcium-induced mPT opening in permeabilized C2C12 cells were higher if ANT1 was knocked down, than scramble RNA treated cells. We therefore conclude that ANT1 is the module conferring sensitivity of the pore to voltage likely through the electrochemical proton gradient across the inner membrane and/or the electron flow though the electron transport chain. This is in line with previous postulations that ANT either exerts an indirect effect on the mPT by influencing surface potential^82^,^83^, or through altered ANT conformation induced by adenine nucleotide binding affected by membrane potential^32^.

## Materials and Methods

### Cell cultures and transfections

Fibroblast cultures from skin biopsies from the patient with no ANT1 expression (AF) the heterozygous mother (ST) and three control subjects were prepared. All experiments were conducted in accordance with the guidelines of Ethical Rules for Using Human Tissues for Medical Research in Hungary (HM 34/1999) and the Code of Ethics of the World Medical Association (Declaration of Helsinki), and were approved by the Strasbourg University Hospital Ethics Committee. Informed consent was obtained from the patient, her mother, and three healthy donors. Cells were grown on poly-L-ornithine coated 8-well LabTek II chambered coverglasses (Nunc, Rochester, NY, USA) for 2–3 days, at a density of approximately 3*10^5^ cells/well in RPMI1640 medium (GIBCO, Life technologies, Carlsbad, CA, USA) supplemented with 10% fetal bovine serum and 2 mM glutamine and kept at 37 °C in 5% CO_2_. The medium was also supplemented with penicillin, streptomycin and amphotericin (item A5955, Sigma-Aldrich St. Louis, MO, USA). Cultures were transfected on the 1st or 2nd DIV with mito-DsRed2 using Lipofectamine 2000 (Life technologies) in RPMI1640 medium at a 3:2 ratio of Lipofectamine (μl) to plasmid DNA (μg) or with mitochondria targeted FRET-based calcium indicator, 4mtD3cpv using flashFECTIN (Oxford Expression Technologies, Oxford, UK) according to the manufacturer’s instructions. Experiments were carried out at day 1–2 post transfection. Typical transfection rates were <5% and therefore individual, non-overlapping cells were visualized.

### Imaging of cultured cells and estimation of *in situ* mitochondrial swelling with the TR technique

Time lapse epifluorescence microscopy was carried out to image cells expressing mito-DsRed2 at 34 °C and swelling of *in situ* mitochondria was measured by the thinness ratio (TR) technique exactly as described in^44^. Basically, cells were imaged directly on the LabTek chamber without superfusion in a medium containing (in mM): 120 NaCl, 3.5 KCl, 1.3 CaCl_2_, 20 HEPES, 15 glucose (where indicated) at pH 7.4. Experiments were performed on an Olympus IX81 inverted microscope equipped with a 60 × 1.4 NA oil immersion lens, a Bioprecision-2 *xy*-stage (Ludl Electronic Products Ltd., Hawthorne, NY) and a 75W xenon arc lamp (Lambda LS, Sutter Instruments, Novato, CA). For mito-DsRed2 an 535/20 nm exciter, a 555LP dichroic mirror and an 570LP emitter (Omega Optical, Brattleboro, VT) were used. Time lapses of *z*-series of 16 planes of 512 × 512 pixels frames (digitized at 14bit with no binning, 250 ms exposure time, yielding 0.1 μm pixel size and 0.8 μm *z*-spacing) were acquired using an ORCA-ER2 cooled digital CCD camera (Hamamatsu Photonics, Hamamatsu, Japan) under control of MetaMorph 6.0 software (Molecular Devices; Sunnyvale, CA, USA). The TR technique measures changes of average diameters of thread-like or punctate structures in fluorescence images using a pair of (high and low frequency) bandpass spatial filters. A calibration image series of mito-DsRed2 fluorescence showing mitochondrial swelling by valinomycin (200 nM) was recorded and used to train a spatial bandpass filter set in Image Analyst MKII (Image Analyst Software, Novato, CA). To calculate TR, for each time point the z-stack was mean-intensity projected and the projection image was duplicated. Then, both images were spatially filtered and the absolute value of pixels was taken. The TR was calculated as the ratio of the average fluorescence intensity in the high frequency band pass filtered over the low frequency band pass filtered image. Mitochondrial swelling causes the loss of high spatial frequency image details, therefore a decrease in the TR value. Baseline normalized TR is given as δTR = (TR − TR_0_)/TR_0_.

### Mitochondrial Ca^2+^ imaging

Two imaging approaches were used: X-Rhod-1 imaging or mitochondria targeted FRET-based calcium indicator, 4mtD3cpv imaging. In X-Rhod-1 experiments cultured cells were loaded with 4 micromole X-Rhod-1-AM for 15 min at 37 °C, in a medium containing (in mM): 120 NaCl, 3.5 KCl, 1.3 CaCl_2_, 20 HEPES, 15 glucose (where indicated) at pH 7.4, then for 25 min cultures were kept in dark for ‘de-esterification’. Experiments were performed at 34 °C on the setup mentioned above with a 60 × 1.4 NA oil immersion lens. For XRhod1 a 535/20 nm exciter, a 555LP dichroic mirror and a 570 LP emitter were used, all from Chroma Technology Corp., Bellows Falls, VT, USA. Time lapses of *z*-series of 5 planes of 1342 × 1024 pixels frames (digitized at 14bit with no binning, 250 ms exposure time, yielding 0.1 μm pixel size and 1.6 μm *z*-spacing) were acquired. For each time point the z-stack was mean-intensity projected. In 4mtD3cpv experiments cultured cells were imaged in a medium containing (in mM): 120 NaCl, 3.5 KCl, 1.3 CaCl_2_, 20 HEPES, 15 glucose (where indicated) at pH 7.4. Experiments were performed at 34 °C on the setup mentioned above with a 60 × 1.4 NA oil immersion lens. For 4mtD3cpV the following filter sets were used: a 440/20 exciter, a dual bandpass (450–490 nm and 520–560 nm transmitting) dichroic mirror, a 480/10 emitter for CFP (CFP channel) and 535/20 emitter for YFP variant (FRET channel), all from Chroma Technology Corp., Bellows Falls, VT. Time lapses of 1344 × 1024 pixels frames for both channels (digitized at 14 bit with no binning, 400 msec exposure times) were acquired.

### Estimation of onset of mPT with the cobalt-calcein technique

Because transfection efficiency with mito-DsRed2 was low, in addition to the thinness ratio technique we assessed onset of mPT implying *in situ* mitochondrial swelling by the cobalt-calcein method, developed by Petronilli *et al.*^43^. Briefly, cells were loaded with 50 nM calcein-AM (above 100 nM, calcein was toxic for these cells) for 20 min at 4 °C, in a medium containing (in mM): 120 NaCl, 3.5 KCl, 1.3 CaCl_2_, 20 HEPES, 15 glucose (where indicated) at pH 7.4. Subsequently, 1 mM CoCl_2_ was added, and the LabTek chamber was mounted on the microscope stage. Experiments were performed at 34 °C on the setup mentioned above with a 60 × 1.4 NA oil immersion lens. For calcein an 488/6 nm exciter, a 505LP dichroic mirror and an 535/25 emitter (all from Chroma Technology Corp., Bellows Falls, VT) were used. Time lapses of *z*-series of 5 planes of 1342 × 1024 pixels frames (digitized at 14 bit with no binning, 250 ms exposure time, yielding 0.1 μm pixel size and 1.6 μm *z*-spacing) were acquired. For each time point the z-stack was mean-intensity projected.

### Simultaneous measurement of mitochondrial (ΔΨm) and plasma membrane potential (PMP)

Cultures were incubated at 37°C in imaging medium containing (in mM): 120 NaCl, 3.5 KCl, 1.3 CaCl_2_, 20 HEPES, 15 glucose (where indicated) at pH 7.4 with TMRM (180 nM) plus the bis-oxonol type plasma membrane potential indicator, DIBAC_4_(3) (250 nM, Life Technologies Inc.) for 60 min before the experiment. Experiments were performed at 34 °C on the setup mentioned above with a UAPO 20× air 0.75NA lens. Time lapses of 1342 × 1024 pixels frames (digitized at 12 bit with 4 × 4 binning, 250 msec exposure time for TMRM and 100 msec exposure time for DIBAC_4_(3) were acquired. For the illumination of DIBAC_4_(3) a 490/10 nm exciter, a 505LP dichroic mirror and 535/25 emission filter were used, for TMRM a 535/20 nm exciter, a 555LP dichroic mirror and a 630/75 emitter were used, all from Chroma Technology Corp., Bellows Falls, VT. The cross-talk of TMRM and DIBAC_4_(3) emissions was eliminated by a linear spectral un-mixing algorithm implemented in Image Analyst MKII (Image Analyst Software, Novato, CA) as previously described^51^, using decomposition algorithms developed in^52^. The algorithm calculates un-mixed fluorescence intensities by solving a linear equation with the cross-talk coefficient matrix. Coefficient matrix was determined by loading the cultures either with TMRM or DIBAC_4_(3) while both emissions (TMRM and DIBAC_4_(3)) were recorded, see Supplementary Fig. 1. At the end of every experiment, full mitochondrial depolarization and plasma membrane depolarization was achieved by the application of mitochondrial (MDC) and plasma membrane depolarization cocktails (CDC). MDC contained (in μM): 1 valinomycin, 1 SF 6847, 2 oligomycin; CDC contained (in μM): 1 valinomycin, 1 SF 6847, 2 oligomycin, 10 nigericin, 10 monensin.

### Northern blotting

Total RNA was isolated using Trizol reagent (Life technologies). RT-PCR was performed with the Phusion RT-PCR kit (Thermo Scientific, Waltham, MA, USA) according to the manufacturer’s instructions. Primers (Sigma-Aldrich, St. Louis, MO, USA) were designed using Primer 3^84^, and were identical to those used by Le Bras *et al.*^85^. The primers were the following: hANT1-F: ATGGGTGATCACGCTTGGAGCTTCCTAAAG and hANT1-R: TTAGACATATTTTTTGATCTCATCATACAA, hANT2-F: cagcagtctgcctcctcttt and hANT2-R: aagctttgcctccttcatca.

### qPCR

Total RNA from the fibroblast cultures was isolated using Trizol reagent (Life technologies). cDNA was synthesized with Oligo d(T)23 VN primer (New England Biolabs, Ipswich, MA, USA) and M-MuLV Reverse Transcriptase (New England Biolabs) as recommended by the manufacturer. Real-time PCR was performed using Power SYBR PCR Mastermix (Life Technologies) and ABI 7900HT Fast Real Time PCR cycler. Primer sequences were obtained from PrimerBank (http://pga.mgh.harvard.edu/primerbank/index.html): ATCACGCTTGGAGCTTCCTAA - ANT1-F, TGCTTCTCAGCACTGATCTGT – ANT1-R; TTATAGACTGCGTGGTCCGTA – ANT2-F; GGCGAAGTTAAGAGCCTGGG – ANT2-R. The results were normalized with the housekeeping human β-actin gene (primers: CTGGCACCCAGCACAATG– HsACTB-F; CCGATCCACACGGAGTACTTG– HsACTB-R^86^). The data were obtained from 3 independent experiments.

### Ant1-silenced C2C12 stable cells

Mouse Ant1 in C2C12 cells was silenced with MISSION shRNA lentiviral transduction particles (Sigma, St Louis, MO), as described previously^56^. Briefly, stable cells were created based on the manufacturer’s protocol in C2C12 myoblasts and selected with 1 mg/ml puromycin. Scrambled shRNA lentivirus was used to create the control cell line.

### Harvesting and permeabilization of cells for ADP-ATP exchange rate measurements and determination of membrane potential

Approximately 1 million fibroblasts (per experiment) cultured as above in poly-L-ornithine coated T-175 cm^2^ flasks (Nunc, Rochester, NY, USA) were washed once in phosphate-buffered saline and harvested with 1 ml of 0.25% trypsin-EDTA, inactivated by 9 ml calf serum, followed by centrifugation at 1,100 g for 2 minutes. Next, cells were washed once in ice-cold buffer containing, in mM: KCl 8, K-gluconate 110, NaCl 10, Hepes 10, KH_2_PO_4_ 10, EGTA 0.005, mannitol 10, MgCl_2_ 1, and 0.5 mg/ml bovine serum albumin (fatty acid-free), pH 7.25 without disturbing the pellet. After the wash, cells were resuspended in 0.2 ml of the same buffer but pre-warmed to 37 °C and also including mitochondrial substrates (5 mM glutamate and 5 mM malate), 25 μM sodium orthovanadate, beryllium trifluoride (see below regarding final concentration) and 50 μM digitonin. Both ADP-ATP exchange rate- and ΔΨm measurements of *in situ* mitochondria in permeabilized cells occurred in this buffer. The rationale for using this particular buffer composition is elaborated in^87^^,^^88^. Glutamate and malate were chosen as mitochondrial substrates because they support mitochondrial substrate-level phosphorylation, and as such, they contribute to greater ATP efflux rates^46^,^49^,^50^.

### [Mg^2+^]_free_ determination from Magnesium Green fluorescence in the experimental volume containing permeabilized fibroblasts and conversion to ADP-ATP exchange rate

ADP-ATP exchange rate was estimated using the recently described fluorimetric method by our laboratory^87^, exploiting the differential affinity of ADP and ATP to Mg^2+^, with the modifications required for digitonin-permeabilized cells, using sodium orthovanadate and beryllium trifluoride, see below and ref. 88. The rate of ATP appearing in the medium following addition of ADP (2 mM) to energized *in situ* mitochondria was calculated from the measured rate of change in free extramitochondrial [Mg^2+^] using standard binding equations. [Mg^2+^] in the extramitochondrial environment was measured using the fluorescent indicator, Magnesium Green 5K^+^ salt (1 μM). The assay is designed such that the ANT is the sole mediator of changes in [Mg^2+^]_free_ in the extramitochondrial volume, as a result of ADP-ATP exchange. For the calculation of [ATP] or [ADP] from [Mg^2+^]_free_, the apparent K_*d*_ values are identical to those in (26) due to identical experimental conditions (K_*ADP*_ = 0.906 ± 0.023 mM, and K_*ATP*_ = 0.114 ± 0.005 mM). Magnesium Green fluorescence was recorded in Tecan Infinite® 200 PRO series plate reader (Tecan Deutschland GmbH, Crailsheim, Germany) at a 0.5 Hz acquisition rate, using 505 and 535 nm excitation and emission wavelengths, respectively. Experiments were performed at 37 °C.

### Mitochondrial membrane potential (ΔΨm) determination of *in situ* mitochondria of permeabilized cells

ΔΨm was estimated using fluorescence quenching of the cationic dye safranin O due to its accumulation inside energized mitochondria^46^, also taking into account the considerations discussed in^89^ and^90^. Briefly, ~30,000 C2C12 cells (per experiment) were cultured in 35 mm wells or ~1 million fibroblasts (per experiment) cultured in poly-L-ornithine coated T-175 cm^2^ flasks were washed once in phosphate-buffered saline and harvested with 0.25% trypsin-EDTA, inactivated by calf serum, followed by centrifugation at 1,100 g for 2 minutes. Next, cells were washed once in an ice-cold buffer containing, in mM: KCl 8, K-gluconate 110, NaCl 10, Hepes 10, KH_2_PO_4_ 10, EGTA 0.005, mannitol 10, MgCl_2_ 1, and 0.5 mg/ml bovine serum albumin (fatty acid-free), pH 7.25 without disturbing the pellet. After the wash, cells were resuspended in 0.2 ml of the same buffer but pre-warmed to 37 °C and also including mitochondrial substrates (5 mM glutamate and 5 mM malate), 5 μM safranin O and 50 μM digitonin. For results obtained from fibroblasts, traces obtained from safranin O fluorescence were calibrated to millivolts as described in ref. 50. For results obtained from C2C12 cells, traces are shown as arbitrary units of safranine O fluorescence. Fluorescence was recorded in a Tecan Infinite® 200 PRO series plate reader at a 0.08 Hz acquisition rate, using 495 and 585 nm excitation and emission wavelengths, respectively. After the completion of each experiment, cells were harvested from each well and protein content was determined. Only those data were considered in which protein content variability among wells was <5%. All experiments were performed at 37 °C and in triplicates.

### Mitochondrial calcium uptake capacity in permeabilized C2C12 cells

Maximum Ca^2+^ uptake capacity of *in situ mitochondria* of permeabilized cells was estimated as detailed previously^57^, with minor modifications: Briefly, approximately 30,000 C2C12 cells (per experiment) were cultured in 35 mm wells, washed once in phosphate-buffered saline and harvested with 0.2 ml of 0.25% trypsin-EDTA, inactivated by 1.8 ml calf serum, followed by centrifugation at 1,100 g for 2 minutes. Next, cells were washed once in an ice-cold buffer containing, in mM: KCl 8, K-gluconate 110, NaCl 10, Hepes 10, KH_2_PO_4_ 10, EGTA 0.005, mannitol 10, MgCl_2_ 1, and 0.5 mg/ml bovine serum albumin (fatty acid-free), pH 7.25 without disturbing the pellet. After the wash, cells were resuspended in 0.2 ml of the same buffer, but pre-warmed to 37 °C and also including mitochondrial substrates (5 mM glutamate and 5 mM malate), 1 μM Calcium Green 5N hexapotassium salt, (CaGr, Life Technologies) and 50 μM digitonin. Consecutive additions of CaCl_2_ was added (100 μM boluses) or NaCN (50 μM boluses) or SF6847 (10 or 100 nM boluses) where indicated, until there was no further decrease in CaGr fluorescence, or upon reaching maximum depolarization inferred by parallel measurements of mitochondrial membrane potential measured by safranine O as detailed above. Fluorescence was recorded in a Tecan Infinite® 200 PRO series plate reader at a 0.08 Hz acquisition rate, using 506 and 531 nm excitation and emission wavelengths, respectively. All experiments were performed at 37 °C and in triplicates. After the completion of each experiment, cells were harvested from each well and protein content was determined. Only those data were considered in which protein content variability among wells was <5%.

### Western blot analysis

C2C12 cells or human fibroblasts were harvested by trypsinization, washed in phosphate-buffered saline, solubilised in RIPA buffer containing a cocktail of protease inhibitors (Protease Inhibitor Cocktail Set I, Merck Millipore, Billerica, MA, USA) and frozen at −80 °C for further analysis. Frozen pellets were thawed on ice, their protein concentration was determined using the bicinchoninic acid assay as detailed below and separated by sodium dodecyl sulfate – polyacrylamide gel electrophoresis (SDS-PAGE). Separated proteins were transferred to a methanol-activated polyvinylidene difluoride membrane. Immunoblotting was performed as recommended by the manufacturers of the antibodies. Rabbit polyclonal anti-ANT1 (Abcam, Cambridge, UK 1:500 dilution), rabbit polyclonal anti-ANT2 (Biogenesis, UK, 1:500 dilution), rabbit monoclonal anti-VDAC1 (Abcam, 1:5,000 dilution) and mouse monoclonal anti-β actin (Abcam, 1:5,000 dilution) primary antibodies were used. Immunoreactivity was detected using the appropriate peroxidase-linked secondary antibody (1:5,000, donkey anti-rabbit or donkey anti-mouse, Jackson Immunochemicals Europe Ltd, Cambridgeshire, UK) and enhanced chemiluminescence detection reagent (ECL system; Amersham Biosciences GE Healthcare Europe GmbH, Vienna, Austria).

### Cytochrome c release

Permeabilized C2C12 cells undergoing calcium uptake capacity determination using CaGr as detailed above were collected at specified time points, and centrifuged at 21,000 g for 3 min. The supernatants were carefully removed, and both the supernatant and pellet fractions were immediately frozen and stored at −20 °C. Cytochrome c immunoreactivity was quantified in both fractions using an enzyme-linked immunosorbent assay kit (Abcam). Before measurement, the supernatant and pellet samples were diluted 1:20 and 1:200, respectively. The release of cytochrome c from *in situ* mitochondria is expressed as the content of cytochrome c in the supernatant as a percentage of the total content of cytochrome c present in the supernatant plus pellet.

### Preparation of sodium orthovanadate (Na_3_VO_4_) and beryllium trifluoride (BeF_3_^−^)

A 25 mM Na_3_VO_4_ solution was prepared in distilled water (>17 mOhm resistance). The pH of the solution was adjusted to 8.7 with HCl, upon which it turns yellow. This solution was boiled until it turns colorless and cooled to room temperature. The pH was reassessed, and readjusted to pH 8.7 with HCl, upon which the solution turned yellow again. This cycle of boiling until colorless and readjusting the pH was repeated until the solution remains colorless at pH 8.7. Finally, the solution was brought up to the initial volume with distilled water and stored in aliquots at −80 °C. This treatment removes all decavanadate ions present in the Na_3_VO_4_ solution, which induces mitochondrial membrane depolarization and inhibition of oxygen consumption^91^. The final concentration of orthovanadate used was 12.5 μM. Orthovanadate inhibits the oxidation of only disrupted mammalian mitochondria^92^. Likewise, fluoroberyllium nucleoside diphosphate complexes inhibit only the exposed F_1_F_0_-ATPase^93^. BeSO_4_ and NaF are prepared as aqueous solutions of 0.2 M and 0.5 M, respectively, and kept at +4 °C for several years. 0.2 mM BeF_3_^−^ is formed immediately in solution upon mixing of BeSO_4_ (0.2 mM) and NaF (5 mM). Vanadate, beryllium and fluoride salts are highly toxic to tissues and to the environment, and thus require proper handling and disposal. The combination of orthovanadate and BeF_3_^−^ inhibits kinases, mutases, phosphatases, and ATPases^94^,^95^. However, some kinases, such as pyruvate kinase, will remain uninhibited^96^. In this respect, upon permeabilization of the cells one has to totally separate pyruvate kinase from its substrate, phosphoenol pyruvate, i.e. there must be no glucose present in the medium prior to permeabilization, and a few minutes lag time must be allowed prior to ADP-ATP exchange rate measurements in order for the remaining reactions by kinases to ‘die-out’.

### Protein determination

Protein content was measured by the bicinchoninic acid assay^97^ using bovine serum albumin protein as standards and calibrating by a 3 parameter power function, f = y0 + a*xb, where y0 is background absorbance in the absence of protein, a and b are constants, and x is the amount of protein in the unknown samples.

### Reagents

Standard laboratory chemicals, safranine O and digitonin were from Sigma. Calcium Green 5N 6K^+^ salt, Magnesium Green 5K^+^ salt and calcein-AM were from Life technologies. SF 6847 was from Biomol (BIOMOL GmbH, Hamburg, Germany). Fetal bovine serum was from Atlanta Biologicals (Lawrenceville, GA, USA) and all other tissue culture reagents were purchased from Life technologies. pDsRed2-mito (mito-DsRed2) was purchased from Clontech (Mountain View, CA). pcDNA-4mtD3cpv was a gift from Amy Palmer & Roger Tsien (Addgene plasmid # 36324).

### Statistics

Data are presented as mean ± SEM; significant differences between two groups of data were evaluated by Student’s t-test, with p < 0.05 considered significant. Significant differences between three or more groups of data were evaluated by one-way analysis of variance followed by Tukey’s *post-hoc* analysis, with p < 0.05 considered statistically significant. If normality test failed, ANOVA on Ranks was performed.

## Additional Information

**How to cite this article**: Doczi, J. *et al.* Alterations in voltage-sensing of the mitochondrial permeability transition pore in ANT1-deficient cells. *Sci. Rep.* **6**, 26700; doi: 10.1038/srep26700 (2016).

## Supplementary Material

Supplementary Information

Supplementary video 1

Supplementary video 2

Supplementary video 3

Supplementary video 4

Supplementary video 5

Supplementary video 6

Supplementary video 7
