# Supplementary material for: Alterations in voltage-sensing of the mitochondrial permeability transition pore in ANT1-deficient cells
Source: Sci Rep. 2016 May 25;6:26700. doi: 10.1038/srep26700 (PMC4879635; doi:10.1038/srep26700)
Supplement: Supplementary Information [file srep26700-s1.pdf]

Supplemental Material for:

Alterations in voltage-sensing of the mitochondrial permeability transition pore in ANT1-deficient cells

RUNNING TITLE: ANT1 and voltage sensing of mPT

Judit Doczi[1,2], Beata Torocsik[1], Andoni Echaniz-Laguna[3], Bénédicte Mousson de Camaret[4], Anatoly Starkov[5], Natalia Starkova[6], Aniko Gál[7], Mária J Molnár[7], Hibiki Kawamata[5], Giovanni Manfredi[5], Vera Adam-Vizi[1] and Christos Chinopoulos[1,2]

[1]Department of Medical Biochemistry, Semmelweis University, Budapest, 1094, Hungary, MTA-SE Laboratory for Neurobiochemistry
[2]MTA-SE Lendület Neurobiochemistry Research Group
[3]Département de Neurologie, Hôpitaux Universitaires, Hôpital de Hautepierre, 67098 Strasbourg cedex, France
[4]Service des Maladies Héréditaires du Métabolisme, Centre de Biologie et de Pathologie Est, CHU Lyon, 69677 Bron cedex, France
[5]Feil Family Brain and Mind Research Institute, Weill Cornell Medical College, New York, NY 10065, USA
[6]Icahn School of Medicine at Mount Sinai, Department of Hematology and Medical Oncology, New York, NY 10029, USA
[7]Institute of Genomic Medicine and Rare Disorders, Semmelweis University, Budapest, 1083, Hungary

To whom correspondence should be addressed: Christos Chinopoulos, Department of Medical Biochemistry, Semmelweis University, Budapest, Tuzolto st. 37-47, 1094, Hungary, Tel: +361 4591500 ext. 60024; Fax: +361 2670031; E-mail: chinopoulos.christos@eok.sote.hu

LEGENDS TO SUPPLEMENTAL FIGURES:

Supplemental Figure 1: Determination of cross-talk of TMRM fluorescence on DIBAC(4)3 channel detection and vice versa. Fibroblasts were loaded with either TMRM only (left side), DIBAC4(3) only (middle), or TMRM plus DIBAC4(3) (right side). In each occasion, time-lapse images were recorded in the TMRM channel and DIBAC4(3) channel, while cells were subjected to 10 μM diazoxide establishing the K^+ equilibrium across the plasma membrane, followed by stepwise increments of extracellular $[K^+]$ (exact values indicated on the left side of the panels). Finally, a cell membrane calibration cocktail (CDC) and a mitochondrial membrane depolarization cocktail (MDC) were applied. Averaged fluorescence values of regions of interest (ROIs) placed on the cells are shown in main figure panels 4B and 4C. From these experiments we determined the cross-talk coefficient matrix which was used to perform the spectral un-mixing of TMRM and DIBAC4(3) fluorescence, shown in supplemental figure 2.

Supplemental Figure 2: Imaging work-flow for spectral un-mixing of TMRM and DIBAC4(3) fluorescence in co-loaded cells using Image Analyst and the cross-talk coefficient matrix determined in the experiments outlined in supplemental figure 1; the effects of maximum loss of $\Delta\Psi p$ and $\Delta\Psi m$ by application of CDC and MDC are shown in the bottom panels.

LEGENDS TO VIDEOS:

Video "mPT_DsRed2_control_HF_calcimycin_plus_Glc": Time-lapse series of a human fibroblast transfected with DsRed2 undergoing mPT by calcimycin treatment, in the presence of glucose in the media.

Video "mPT_DsRed2_control_HF_calcimycin_no_Glc_plus_CN": Time-lapse series of a human fibroblast transfected with DsRed2 undergoing mPT by calcimycin treatment, in the absence of glucose, and presence of 2-DG and NaCN in the media.

Video "mPT_DsRed2_control_HF_calcimycin_no_Glc_plus_UNC": Time-lapse series of a human fibroblast transfected with DsRed2 undergoing mPT by calcimycin treatment, in the absence of glucose, and presence of 2-DG and SF6847 in the media.

Video "mPT_COBCA_control_HF_calcimycin_plus_Glc": Time-lapse series of human fibroblasts loaded with calcein while the cytosolic signal was quenched by cobalt (cobalt-calcein technique) undergoing mPT by calcimycin treatment, in the presence of glucose in the media.

Video "(mPT_DsRed2_control_HF_H2O2": Time-lapse series of a human fibroblast transfected with DsRed2 undergoing mPT by H_2O_2 treatment, in the presence of glucose in the media.

Video "mPT_DsRed2_C2C12_shRNA_ANT1": Time-lapse series of a C2C12 myotube transfected with shRNA directed against ANT1 with lentiviruses and DsRed2 undergoing mPT by calcimycin treatment, in the presence of glucose in the media.

Video "mPT_DsRed2_C2C12_scramble_ANT1": Time-lapse series of a C2C12 myotube transfected with scramble RNA with lentiviruses and DsRed2 undergoing mPT by calcimycin treatment, in the presence of glucose in the media.

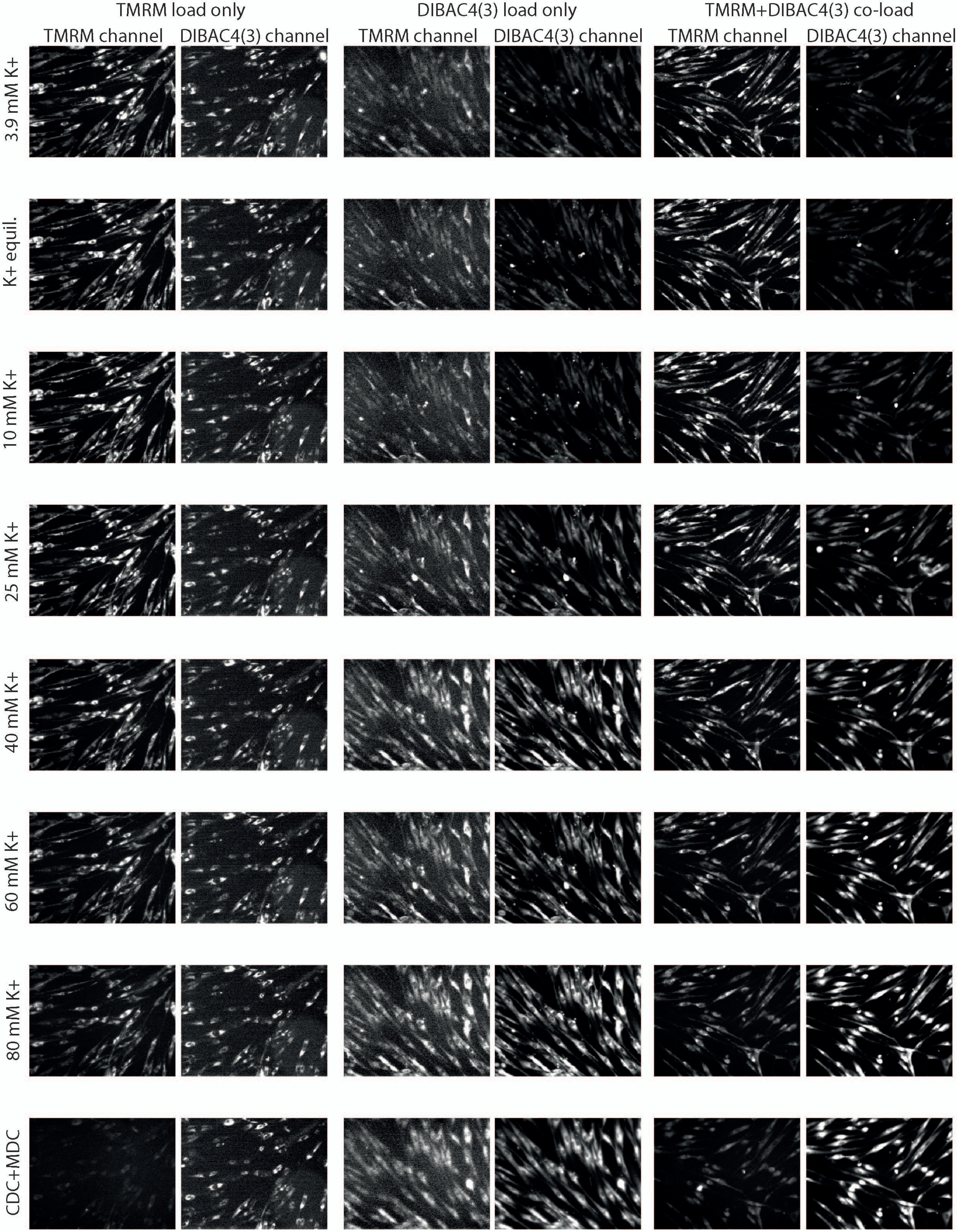

Supplemental Figure 1

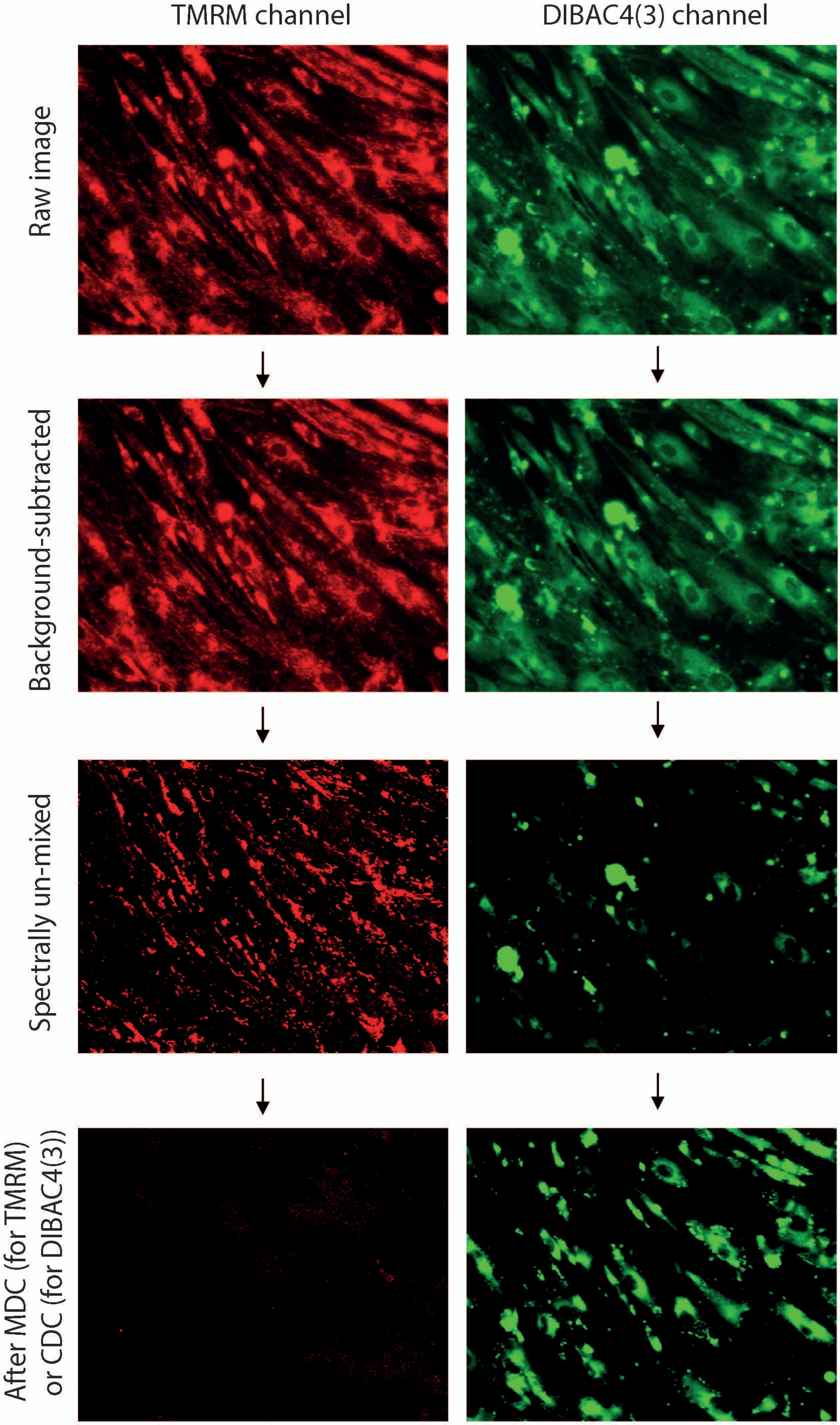

Supplemental Figure 2
